# Non-invasive oxygenation support in acutely hypoxemic COVID-19 patients admitted to the ICU: a multicenter observational retrospective study

**DOI:** 10.1186/s13054-022-03905-5

**Published:** 2022-02-08

**Authors:** Pedro David Wendel-Garcia, Arantxa Mas, Cristina González-Isern, Ricard Ferrer, Rafael Máñez, Joan-Ramon Masclans, Elena Sandoval, Paula Vera, Josep Trenado, Rafael Fernández, Josep-Maria Sirvent, Melcior Martínez, Mercedes Ibarz, Pau Garro, José Luis Lopera, María Bodí, Joan Carles Yébenes-Reyes, Carles Triginer, Imma Vallverdú, Anna Baró, Fernanda Bodí, Paula Saludes, Mauricio Valencia, Ferran Roche-Campo, Arturo Huerta, Francisco José Cambra, Carme Barberà, Jorge Echevarria, Óscar Peñuelas, Jordi Mancebo, for the UCIsCAT study group, R. Ferrer, O. Roca, X. Nuvials, J. C. Ruiz, E. Papiol, R. Máñez, V. D. Gumicio, E. Sandoval, G. Muñoz, D. Toapanta, P. Castro, J. Osorio, J. R. Masclans, R. Muñoz-Bermúdez, F. Parrilla, P. Pérez-Teran, J. Marin-Corral, A. Mas, B. Cancio, S. Hernández-Marín, M. R. Koborzan, C. A. Briones, J. Trenado, R. Fernández, J. M. Sirvent, P. Sebastian, X. Saiz, M. Martínez, M. Ibarz, P. Garro, C. Pedrós, E. Vendrell, J. L. Lopera, M. Bodí, A. Rodríguez, G. Moreno, J. C. Yébenes-Reyes, C. Triginer, I. Vallverdú, A. Baró, M. Morales, F. Bodí, P. Saludes, J.-R. Cervelló, M. Valencia, F. Roche-Campo, D. Franch-Llasat, A. Huerta, P. Santigosa, F. J. Cambra, S. Benito, C. Barberà, J. Echevarría, J. Mancebo, P. Vera, J.-A. Santos, J. Baldirà, A.-J. Betbesé, M. Izura, I. Morán, J.-C. Suárez, L. Zapata, N. Rodríguez, M. Torrens, A. Cordón, C. Gomila, M. Flores, A. Segarra, M. Morales, L. Mateo, M. Martos, C. González-Isern

**Affiliations:** 1 Institute of Intensive Care Medicine, University Hospital of Zurich, Zurich, Switzerland; 2 Intensive Care Department, Hospital de Sant Joan Despí Moisès Broggi, Sant Joan Despí, Spain; 3 Medical Technology Department, Hospital de La Santa Creu I Sant Pau, Barcelona, Spain; 4 Intensive Care Department/SODIR Research Group, Hospital Universitari General de La Vall d’Hebron, Barcelona, Spain; 5 Intensive Care Department, L’Hospitalet de Llobregat, Barcelona, Spain; 6 Intensive Care Department, Hospital del Mar, GREPAC Research Group - IMIM, Department Ciències, Experimentals I de La Salut (DCEXS) UPF, Barcelona, Spain; 7 Cardiovascular Surgery Department, Hospital Clínic de Barcelona, Barcelona, Spain; 8 Intensive Care Department, Hospital de La Santa Creu I Sant Pau, Barcelona, Spain; 9 Intensive Care Department, Hospital Mútua de Terrassa, Terrassa, Spain; 10 Intensive Care Department, Althaia, Xarxa Assistencial Universitària de Manresa, Manresa, Spain; 11 Intensive Care Department, Hospital Universitari Doctor Josep Trueta de Girona, Girona, Spain; 12 Intensive Care Department, Hospital General De Cataluña, Sant Cugat del Vallès, Spain; 13 Intensive Care Department, Hospital Universitari Sagrat Cor - Grup Quirónsalut, Barcelona, Spain; 14 Intensive Care Department, Hospital General de Granollers, Granollers, Spain; 15 Intensive Care Department, Hospital General de Vic, Consorci Hospitalari de Vic, Vic, Spain; 16 Intensive Care Department, Hospital Universitari de Tarragona Joan XXIII, Tarragona, Spain; 17 Intensive Care Department, Hospital de Mataró, Mataró, Spain; 18 Intensive Care Department, Hospital d’Igualada, Igualada, Spain; 19 Intensive Care Department, Hospital Sant Joan de Reus, Reus, Spain; 20 Intensive Care Department, Hospital de Santa Caterina, Salt, Spain; 21 Intensive Care Department, Hospital de Sant Pau I Santa Tecla, Tarragona, Spain; 22 Intensive Care Department, Hospital HM Delfos, Barcelona, Spain; 23 Intensive Care Department, Hospital El Pilar - Grup Quirónsalut, Barcelona, Spain; 24 Intensive Care Department, Hospital de Tortosa Verge de La Cinta, Tortosa, Spain; 25 Intensive Care Department, Clínica Sagrada Família, Barcelona, Spain; 26 Pediatric Intensive Care Department, Hospital Sant Joan de Déu de Barcelona, Esplugues de Llobregat, Spain; 27 Intensive Care Department, Hospital Santa Maria, Lleida, Spain; 28 Intensive Care Department, Hospital ASEPEYO de Barcelona, Sant Cugat del Vallés, Spain; 29 Intensive Care Department Hospital, Universitario de Getafe, CIBER Enfermedades Respiratorias, CIBERES (Spain), Madrid, Spain; 30 Institut d, Investigació Biomèdica Sant Pau, ’, Servei Medicina Intensiva, Hospital Universitari Sant Pau, Barcelona, Spain; 31 Hospital Universitari General de La Vall d’Hebron, Barcelona, Spain; 32 Hospital Universitari de Bellvitge, L’Hospitalet de Llobregat, Barcelona, Spain; 33 Hospital Clínic I Provincial de Barcelona, Barcelona, Spain; 34 Hospital del Mar, Barcelona, Spain; 35 Hospital de Sant Joan Despí Moisès Broggi, Sant Joan Despí, Spain; 36 Hospital Mútua de Terrassa, Terrassa, Spain; 37 Althaia (Xarxa Assistencial Universitària de Manresa), Manresa, Spain; 38 Hospital Universitari Doctor Josep Trueta de Girona, Girona, Spain; 39 Hospital General De Cataluña, Sant Cugat del Vallès, Spain; 40 Hospital Universitari Sagrat Cor - Grup Quirónsalut, Barcelona, Spain; 41 Hospital General de Granollers, Granollers, Spain; 42 Hospital General de Vic (Consorci Hospitalari de Vic), Vic, Spain; 43 Hospital Universitari de Tarragona Joan XXIII, Tarragona, Spain; 44 Hospital de Mataró, Mataró, Spain; 45 Hospital d’Igualada, Igualada, Spain; 46 Hospital Sant Joan de Reus, Reus, Spain; 47 Hospital de Santa Caterina, Salt, Spain; 48 Hospital de Sant Pau I Santa Tecla, Tarragona, Spain; 49 Hospital HM Delfos, Barcelona, Spain; 50 Hospital El Pilar - Grup Quirónsalut, Barcelona, Spain; 51 Hospital de Tortosa Verge de La Cinta, Tortosa, Spain; 52 Clínica Sagrada Família, Barcelona, Spain; 53 Hospital Sant Joan de Déu, Esplugues de Llobregat, Spain; 54 Hospital Santa María, Lleida, Spain; 55 Hospital ASEPEYO de Barcelona, Sant Cugat del Vallés, Spain; 56 Hospital de La Santa Creu I Sant Pau, Barcelona, Spain

**Keywords:** COVID-19, Intensive care, Non-invasive oxygenation, Acute hypoxemic respiratory failure

## Abstract

**Background:**

Non-invasive oxygenation strategies have a prominent role in the treatment of acute hypoxemic respiratory failure during the coronavirus disease 2019 (COVID-19). While the efficacy of these therapies has been studied in hospitalized patients with COVID-19, the clinical outcomes associated with oxygen masks, high-flow oxygen therapy by nasal cannula and non-invasive mechanical ventilation in critically ill intensive care unit (ICU) patients remain unclear.

**Methods:**

In this retrospective study, we used the best of nine covariate balancing algorithms on all baseline covariates in critically ill COVID-19 patients supported with > 10 L of supplemental oxygen at one of the 26 participating ICUs in Catalonia, Spain, between March 14 and April 15, 2020.

**Results:**

Of the 1093 non-invasively oxygenated patients at ICU admission treated with one of the three stand-alone non-invasive oxygenation strategies, 897 (82%) required endotracheal intubation and 310 (28%) died during the ICU stay. High-flow oxygen therapy by nasal cannula (*n* = 439) and non-invasive mechanical ventilation (*n* = 101) were associated with a lower rate of endotracheal intubation (70% and 88%, respectively) than oxygen masks (*n* = 553 and 91% intubated), *p* < 0.001. Compared to oxygen masks, high-flow oxygen therapy by nasal cannula was associated with lower ICU mortality (hazard ratio 0.75 [95% CI 0.58–0.98), and the hazard ratio for ICU mortality was 1.21 [95% CI 0.80–1.83] for non-invasive mechanical ventilation.

**Conclusion:**

In critically ill COVID-19 ICU patients and, in the absence of conclusive data, high-flow oxygen therapy by nasal cannula may be the approach of choice as the primary non-invasive oxygenation support strategy.

**Supplementary Information:**

The online version contains supplementary material available at 10.1186/s13054-022-03905-5.

## Background

The coronavirus disease 2019 (COVID-19), caused by the severe acute respiratory syndrome coronavirus 2 (SARS-CoV-2), has generated an unprecedented demand for intensive care resources to deliver supportive care using non-invasive oxygenation support and invasive mechanical ventilation [1]. Large studies have provided data regarding baseline clinical characteristics of patients admitted to intensive care units (ICU), the need for invasive mechanical ventilation, and outcomes of mechanically ventilated patients [2–10].

During the pandemic, patients with acute hypoxemic respiratory failure admitted to the ICU and not in need of emergent intubation of the trachea have mainly been treated with non-invasive oxygenation strategies [1]. These methods include standard non-rebreather oxygen masks (oxygen mask), high-flow oxygen therapy administered through large-bore nasal cannulas (HFT), and non-invasive positive pressure ventilation (NIV) techniques [1, 2, 11]. The choice for using one type of oxygenation support over another has likely been based on local recommendations, personal experience, and availability of devices.

The difference of opinions regarding the optimal technique for non-invasive oxygenation support is ample, but the consequences and relevant clinical outcomes of the various usual care strategies in critically ill COVID-19 patients admitted to an ICU remain unclear [12–15]. Thus, our main objective was to evaluate the impact of three stand-alone non-invasive oxygenation strategies on intubation rates and ICU mortality at 90 days after admission of critically ill patients with COVID-19-associated acute hypoxemic respiratory failure. We hypothesized that the various non-invasive oxygenation support strategies had no impact on intubation and ICU mortality rates.

## Methods

We retrospectively analysed a cohort of patients admitted to ICUs in the Spanish autonomous community of Catalonia between March 14 and April 15, 2020. The official census population of Catalonia at the time was 7,780,479 inhabitants. At the start of the pandemic, the Catalonian public health system had approximately 500 adult ICU beds in its 26 hospitals, and 13 private hospitals had an additional 100–120 ICUs beds for adults. An invitation to participate in the study was sent to the head of department at all the intensive care medicine departments in Catalonia at the end of April 2020. In view of the nature of the study, the ethics committee at the coordinating centre (Hospital Universitari de la Santa Creu i Sant Pau, Barcelona) approved the study protocol (UCIS-CAT 20/151 OBS) and waived the need for informed consent. Participating centres complied with all local requirements.

### Inclusion and exclusion criteria

The inclusion criteria were age 18 years or older, patient admitted to an ICU, clinical signs and symptoms compatible with COVID-19 pneumonia, a positive real-time reverse-transcriptase polymerase chain reaction test for SARS-CoV-2 obtained from a nasopharyngeal swab, bilateral infiltrates in the chest X-ray, need for supplemental oxygen to keep arterial oxygen saturation measured with a pulse oximeter (SpO_2_) above 90%, and admission at one of the participating centres’ ICU between March 14 and April 15, 2020. All patients were candidates for intubation and mechanical ventilation at the time of ICU admission. Decisions regarding intubation of the trachea were based on clinical grounds and judgment of the intensivist in charge.

**Fig. 1:**
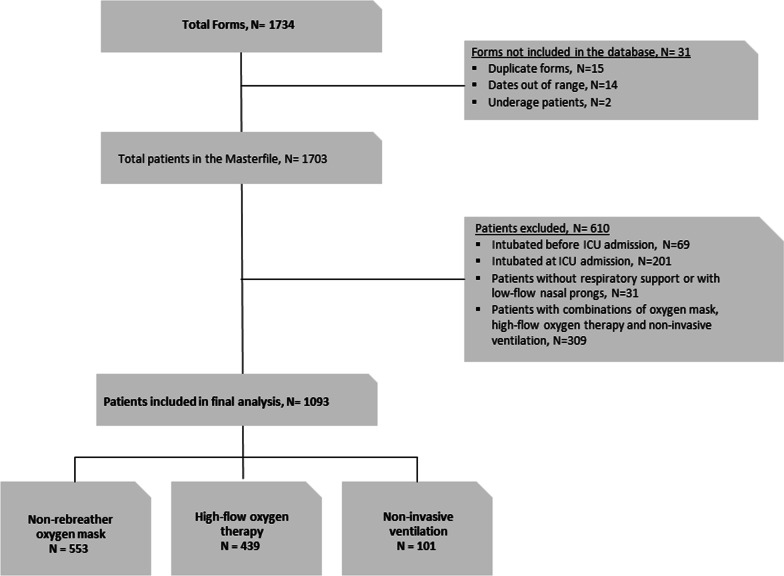
Study flowchart

Only patients who had been treated exclusively with a single non-invasive oxygenation technique during their ICU stay were included in this analysis: oxygen mask at a rate of more than 10 L oxygen/minute, HFT administered through a heated humidifier at a gas flow rate above 30 L/minute and a fraction of inspired oxygen (FiO_2_) of at least 0.5, or NIV with a FiO_2_ of at least 0.5 (irrespective of interface, mode and ventilator type used). The choice of non-invasive oxygenation technique was at the discretion of each participating ICU and was based on local recommendations and availability of devices. The day of ICU admission was defined as day 0. Patients were excluded from our analysis in three situations: first, they were intubated and mechanically ventilated before ICU admission because the context and conditions of intubation were unknown; second, they were intubated immediately after ICU admission (within 3 h) because the attempt at non-invasive oxygenation support was considered clinically unsuccessful; and third, they received oxygen supplementation either by nasal prongs or by any combination of oxygen mask, HFT and NIV (Fig. 1). Patients receiving a combination of multiple non-invasive oxygenation support techniques were not analysed as the order of administration and the reasons for change could not be precisely elucidated, thus introducing an unquantifiable allocation bias into the analysis.

### Data collection

The principal investigator at each centre filled in a standardized case report form for each patient included in the study. Data regarding demographic variables, laboratory data at admission, type of non-invasive and invasive oxygenation support, variables related to gas exchange and respiratory system mechanics obtained shortly after endotracheal intubation, and ICU outcome status at 90 days from ICU admission were processed from the independent case report forms and stored in a single master file. Patients were followed in the ICU until day 90 post-admission or until ICU discharge, whichever occurred first. This file was reviewed independently by three investigators (PDWG, CGI and JM). The principal investigators at each centre were contacted to solve any errors or missing data. Following this process, the master file was closed.

Once intubated, patients who met acute respiratory distress syndrome (ARDS) criteria were classified in mild, moderate and severe oxygenation phenotypes, as described [16]. Driving airway pressure was calculated as the difference between plateau airway pressure and set positive end-expiratory pressure (PEEP). Compliance of the respiratory system was calculated as tidal volume divided by driving airway pressure. The ventilatory ratio and estimated physiological dead-space fraction were calculated as previously described by Sinha et al. [17] and Morales-Quinteros et al. [18], respectively.

### Missing data

To account for missing data (Additional file 1: Table s1), we performed multiple imputations by fully conditional specification with predictive mean matching under the missing at random assumption [19]. Further details regarding the imputation method used are presented in Additional file 1: Appendix 1, Additional file 1: Fig. s1, Additional file 1: Fig. S2 and Additional file 1: Table s2.

### Statistical analysis

To enable causal inference of the average treatment effect associated with each oxygenation support strategy, we evaluated the baseline covariate balance at ICU admission. Covariate balancing refers to a group of statistical methods used to enable exchangeability of exposed and unexposed subjects to one or multiple treatments in order to minimize confounding. Applying a weight on each individual subject generates a standardized pseudo-population, in which causal treatment effect inference is possible. Nine covariate balancing methods considering all baseline covariates at ICU admission were tested against each other (see Additional file 1: Appendix 2). The best covariate balancing method (standardized mean deviation < 0.1 and variance ratio < 2) was used for all subsequent analyses [20]. A detailed description on the covariate balancing method selection is also shown in Additional file 1: Appendix 2 and Additional file 1: Figs. s3 and s4.

Univariable and multivariable Cox proportional hazard models coupled to the Kaplan–Meier estimator, with and without random frailty terms for between-centre variability effect, were used to analyse the effects of the different oxygenation support strategies on the incidence of intubation and ICU mortality. The effects are represented by hazard ratios and their 95% confidence interval (CI). A time-varying Cox proportional hazards model was used to investigate the effect of time between ICU admission and intubation on 90-day ICU mortality. Patients discharged alive from the ICU were considered to be alive at day 90. Survival distributions among the various oxygenation support strategies were compared using the log-rank test. Proportional hazard assumptions were assessed by inspection of Schoenfeld residuals.

Ventilator-free survival days were defined as the cumulative time in the first 30 days of ICU admission without the need for invasive mechanical ventilation. Patients who died within the first 30 days were assigned 0 ventilator-free days. Population characteristics were compared using analysis of variance or the Kruskal–Wallis test, as appropriate, and the chi-squared test for categorical variables. Statistical analysis was performed through a fully scripted data management pathway using the R environment for statistical computing version 4.1.0. No power calculations were performed due to the observational nature of this cohort study. A two-sided *p* < 0.05 was considered statistically significant. Values are given as medians and [interquartile ranges] or counts and percentages as appropriate.

## Results

Twenty-six centres participated in the study, 20 of the 26 public health system hospitals in Catalonia and 6 of 13 private care centres. The study thus reflects the care provided to patients in 438 of the 620 adult ICU beds available in Catalonia at the beginning of the pandemic, 370 pertaining to the public system and 68 to the private health system (see Additional file 1: Appendix 3).

A total of 1703 critically ill COVID-19 patients were admitted to the participating ICUs between March 14 and April 15, 2020. Figure 1 shows the study flowchart and Additional file 1: Table s3 shows the main characteristics and outcomes for the overall unbalanced population. Three-hundred and nine patients were treated with combinations of non-invasive respiratory support techniques and were excluded from the analysis to allow a clear differentiation between individual therapies. Overall, these patients had a 72% intubation rate (222/309) and the ICU mortality rate up to day 90 post-admission was 27.5% (85/309).

### Population analysed and matching analysis

The final analysis included 1093 patients treated with a non-invasive oxygenation support strategy during their ICU stay: 553 (51%) with oxygen mask, 439 (40%) with HFT, and 101 (9%) with NIV. Their main characteristics and outcomes are shown in Additional file 1: Table s4.

Among the various balancing algorithms tested, we employed the targeted stable balancing weights using the optimization algorithm. This algorithm minimized standard mean deviation (< 0.0001) and variance ratios (≤ 1.7) between oxygenation support strategies for all variables and presented consistent and moderate weights (1 ± 0.35) for all patients (Additional file 1: Fig. s5, Additional file 1: Fig. s6, Additional file 1: Fig. s7).

### Characteristics of the analysed population

Baseline characteristics across oxygenation support strategies were identical after covariate balancing (Table 1). Patients were mainly male (68%) with a median age of 63 [54–70] years. The median time between hospital and ICU admission was 1 day [0–3 days]. The overall intubation rate was 82% (897/1093). A total of 501 out of 553 (91%) patients treated with oxygen mask were eventually intubated and mechanically ventilated as opposed to 307 out of 439 (70%) of those treated with HFT and 89 out of 101 (88%) of those treated with NIV (*p* < 0.001). The number of ventilator-free days was higher in the HFT group than in the oxygen mask and NIV groups (*p* < 0.001).

**Table 1:** Demographics, baseline characteristics at intensive care unit admission and overall outcome obtained after covariate balancing*

	Overall	Oxygen mask	High-flow oxygen therapy	Non-invasive ventilation	*p*	SMD	VR
	*n* = 1093	*n* = 553	*n* = 439	*n* = 1011			
Age (years)	63 [54—70]	64 [54—70]	62 [54—70]	63 [53—69]	0.904	< 0.001	1.11
Sex (female)	354 (32)	179 (32)	142 (32)	33 (32)	1	< 0.001	–
Body mass index (kg·m^−2^)	28 [26–31]	28 [25_-_31]	28 [26–31]	28 [26–31]	0.914	< 0.001	1.23
Time from hospital admission to ICU admission [days]	1 [0—3]	1 [0—3]	1 [0—3]	2 [1–3]	0.553	< 0.001	1.22
Comorbidities, n (%)							
Cardiovascular	520 (48)	263 (48)	209 (48)	48 (48)	1	< 0.001	–
Diabetes	226 (21)	114 (21)	91 (21)	21 (21)			
Cancer	80 (7)	41 (7)	32 (7)	7 (7)			
COPD	79 (7)	40 (7)	32 (7)	7 (7)			
Immunosupression	63 (6)	32 (6)	25 (6)	6 (6)			
Leucocytes (G/l)	9 [6–12]	9 [6–12]	9 [6–12]	9 [7–12]	0.782	< 0.001	1.25
Lymphocytes (G/l)	0.7 [0.5—1.0]	0.7 [0.5—1.0]	0.8 [0.5—1.0]	0.7 [0.5—1.0]	0.551	< 0.001	1.28
Neutrophil/ lymphocyte ratio	10 [7–17]	11 [6–18]	10 [7–17]	10 [7–18]	0.688	< 0.001	1.46
Procalcitonin (μg/l)	0.3 [0.1—0.6]	0.3 [0.1—0.6]	0.3 [0.1—0.6]	0.2 [0.1—0.5]	0.254	< 0.001	1.7
C-reactive protein (mg/l)	113 [23–222]	118 [30—217]	104 [17—227]	114 [30—227]	0.296	< 0.001	1.42
Lactate (mmol/l)	1.4 [1.1–1.9]	1.3 [1.0—1.9]	1.4 [1.1—1.9]	1.4 [1.2—1.8]	0.052	< 0.001	1.47
Interleukin-6 (ng/l)	129 [50–329]	132 [50—304]	118 [60—332]	130 [43—354]	0.855	< 0.001	1.34
D-dimers (μg/l)	1145 [560–2965]	1262 [611–3058]	1040 [494—2760]	1190 [558—3793]	0.07	< 0.001	1.57
Ferritin (μg/l)	1387 [760–2259]	1325 [779—2254]	1465 [742—2215]	1382 [722—2356]	0.91	< 0.001	1.58
Intubation, n (%)	897 (82)	501 (91)	307 (70)	89 (88)	< 0.001		
Length of ICU stay (days)	14 [7–26]	14 [7–26]	13 [7–26]	13 [8–24]	0.958		
Ventilator-free survival^†^ (days)	12 [0—24]	8 [0—23]	15 [0—30]	11 [0—24]	< 0.001		
ICU mortality, n (%)	310 (28)	167 (30)	106 (24)	37 (36)	0.041		

Quantitative data are expressed as median [interquartile range]. *p* values are given for the difference between respiratory strategies. Standardized mean differences (SMD) reflect the maximal mean difference between groups. Variance ratios (VR) reflect the maximal higher-order moments and interactions between groups. COPD—chronic obstructive pulmonary disease; CRP—C-reactive protein; ICU—intensive care unit; PCT—Procalcitonin. † Calculated at 30 days post-intensive care unit admission; patients deceased were assigned 0 ventilator free days

*By applying a weight on each individual subject, a standardized pseudo-population is generated, which slightly differs from the original population (e-Table 4). Causal treatment effect inference is possible in this pseudo-population

**Includes any of the following: arterial hypertension, ischemic heart disease, acute cerebrovascular events

Table 2 shows the characteristics of patients who eventually required intubation and mechanical ventilation, after covariate balancing. The median time from ICU admission to endotracheal intubation was 0 [0–0] days in the oxygen mask group, 0 [0–1] days in the HFT group, and 0 [0–1] days in the NIV group (*p* < 0.001). Among the 897 intubated patients, 824 (92%) had an ARDS and this was moderate-to-severe in 571 (69%).

**Table 2:** Ventilator settings and gas-exchange parameters shortly after intubation and outcome in intubated patients

	Overall *n* = 897	Oxygen mask * n* = 501	High-flow oxygen therapy * n* = 307	Non-invasive ventilation * n* = 89	*p*
Time from ICU admission to intubation [days]	0 [0–1]	0 [0—0]	0 [0—1]	0 [0—1]	< 0.001
ARDS classification^†^, n (%)					
No ARDS	73 (8)	48 (9)	18 (6)	7 (8)	0.264
Mild	253 (28)	151 (30)	84 (27)	18 (20)	
Moderate	451 (50)	238 (48)	159 (52)	54 (61)	
Severe	120 (14)	64 (13)	46 (15)	10 (11)	
FiO_2_ (%)	60 [50—80]	60 [50—80]	60 [50—70]	60 [45—80]	0.444
PaO_2_ (mmHg)	97 [79—126]	98 [80—131]	98 [78—122]	91 [78—113]	0.19
PaO_2_/ FiO_2_ (mmHg)	171 [126—229]	173 [124–238]	174 [127—228]	157 [124—205]	0.487
PaCO_2_ (mmHg)	46 [40–54]	46 [40–54]	47 [40–54]	46 [38—57]	0.993
pH	7.35 [7.29—7.4]	7.35 [7.28—7.4]	7.35 [7.29—7.41]	7.35 [7.28—7.4]	0.57
Respiratory rate (1/min)	22 [20–25]	22 [20–25]	22 [20–25]	22 [20–26]	0.555
Tidal volume/ideal body weight [ml/kg]	6.7 [6.0—7.5]	6.7 [6.0—7.5]	6.7 [6.1—7.6]	6.7 [5.7—7.3]	0.27
PEEP (cmH_2_O)	12 [10–14]	12 [10–14]	12 [10–14]	14 [12–15]	< 0.001
Plateau airway pressure (cmH_2_O)	25 [23–28]	25 [22–28]	25 [23–28]	26 [24–28]	0.13
Driving airway pressure (cmH_2_O)	12 [10–15]	12 [10–15]	12 [10–15]	12 [10–15]	0.745
Compliance rs (ml/cmH_2_O)	35 [28–44]	34 [28–42]	35 [27–45]	33 [27–43]	0.591
Ventilatory ratio *	1.9 [1.5–2.3]	1.9 [1.5–2.3]	1.8 [1.5–2.3]	1.8 [1.4–2.3]	0.837
Estimated physiological dead-space fraction (%) **	54 [42–64]	54 [43–63]	55 [39–64]	51 [43–63]	0.823
Length of mechanical ventilation (days)	14 [7–25]	14 [7–24]	16 [9–26]	12 [7–22]	0.031
Length of ICU stay (days)	17 [10–28]	16 [9–27]	19 [12–31]	15 [9–26]	0.004
ICU mortality, n (%)	297 (33)	164 (33)	96 (31)	37 (41)	0.275

Quantitative data are expressed as median [interquartile range]. *P* values are given for the difference between respiratory strategies. ARDS—acute respiratory distress syndrome; ICU—intensive care unit; PaO_2_—partial pressure of arterial oxygen; FiO_2_—fraction of inspired oxygen; PaCO_2_—partial pressure of arterial carbon dioxide; PEEP—positive end-expiratory pressure; Compliance rs—compliance of the respiratory system

^†^Mild: 200mHg < PaO_2_/FiO_2_ ≤ 300 mmHg; Moderate: 100mHg < PaO_2_/ FiO_2_ ≤ 200 mmHg; Severe: PaO_2_/ FiO_2_ ≤ 100 mmHg

*Calculated according to Sinha. (17), see formula in Additional file 1: Appendix 4

**Calculated according to Morales-Quinteros et al*.* (18), see formula in Additional file 1: Appendix 4

**Fig. 2:**
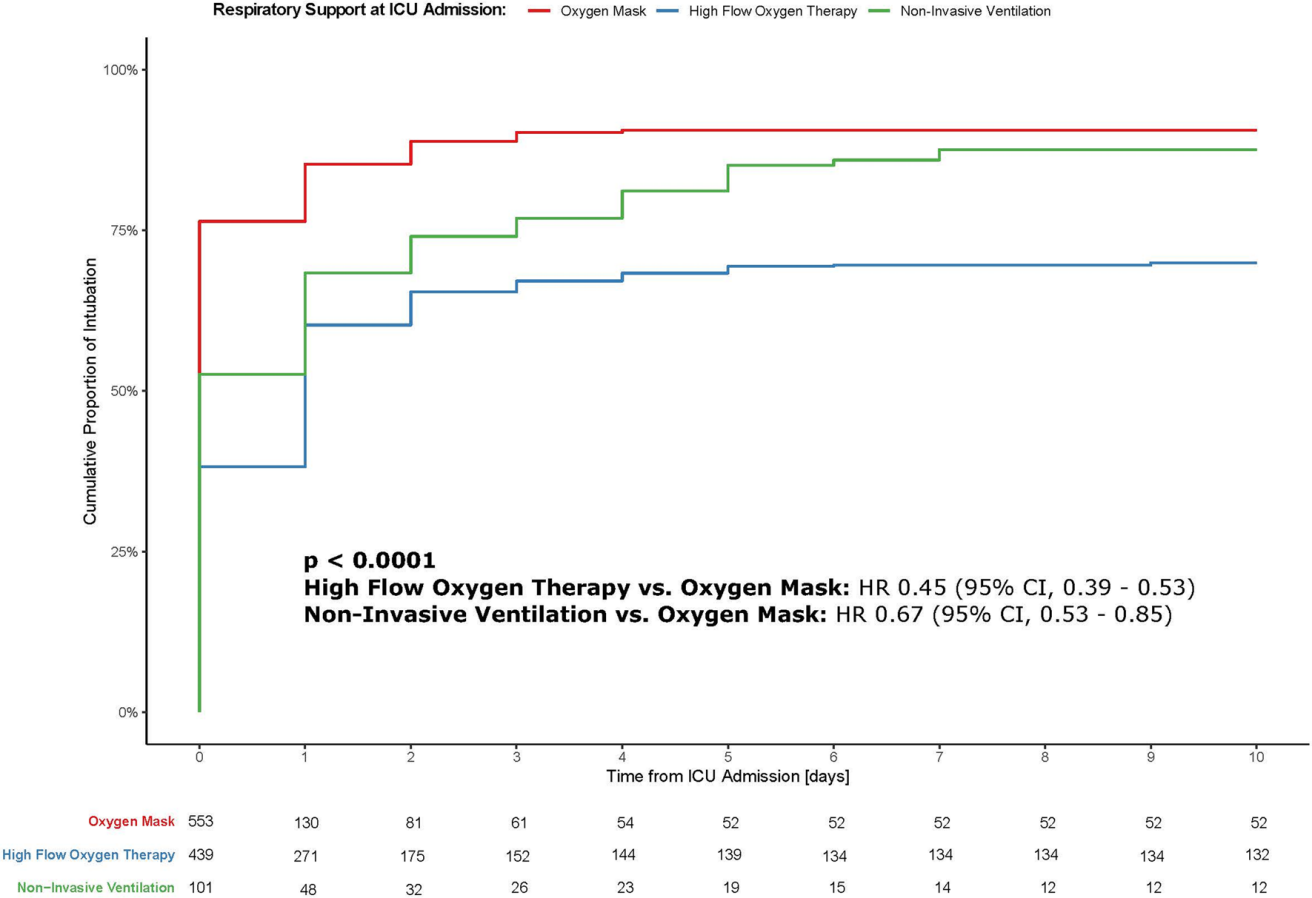
Kaplan–Meier plot of the cumulative proportion of intubation. Kaplan–Meier curve of the cumulative proportion of intubation stratified according to the initial oxygenation support strategy at ICU admission. Oxygen mask, high-flow oxygen therapy and non-invasive ventilation are plotted in red, blue, and green, respectively. p values were calculated by means of the log-rank test. Hazard ratios (HR) were computed using a Cox proportional hazard model and the risk of intubation in the high-flow oxygen therapy and non-invasive ventilation groups was assessed using the oxygen mask group as reference; 95% confidence intervals (CI) are given in parentheses. Table at the bottom presents the patients at risk per time point

Figure 2 shows the cumulative proportion of intubation over time according to the various non-invasive oxygenation support techniques. As compared to oxygen mask, the hazard ratio for intubation in HFT treated patients was 0.45 (95% CI 0.39–0.53) and 0.67 (95% CI 0.53–0.85) for NIV treated patients.

### Mortality

**Fig. 3:**
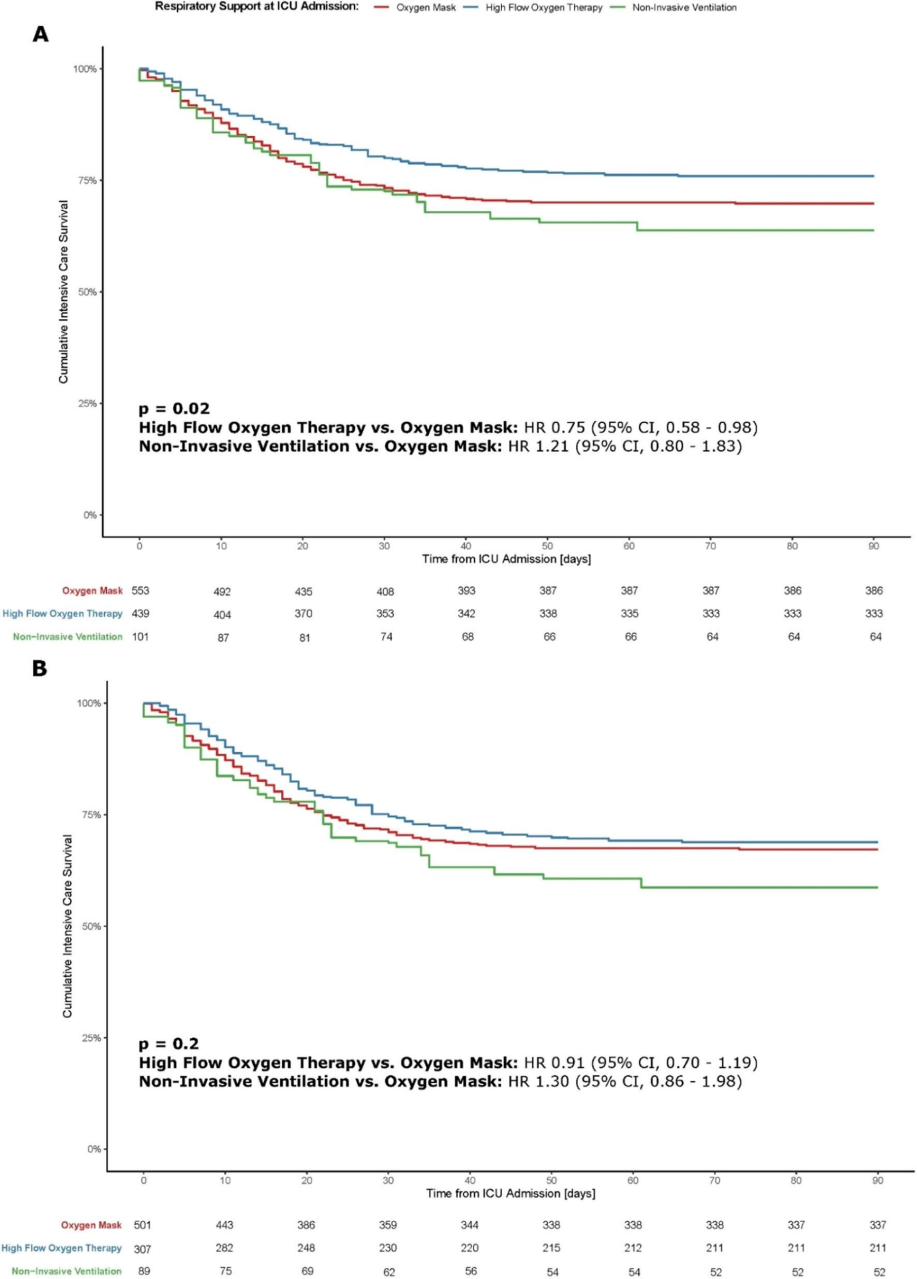
Kaplan–Meier plot of the cumulative survival in the intensive care unit. Kaplan–Meier curve of the cumulative intensive care survival was stratified according to the initial oxygenation support strategy at admission to the intensive care unit. Subplot (**a**) refers to all patients included in the analysis. Subplot (**b**) considers only patients pending probably intubation and invasive mechanical ventilation. Oxygen mask, high-flow oxygen therapy and non-invasive ventilation are plotted in red, blue, and green, respectively. *p* values were calculated by means of the log-rank test. Hazard ratios (HR) were computed by means of a Cox proportional hazard model. The risk of intensive care unit mortality in the high-flow oxygen therapy and non-invasive ventilation groups was assessed using the oxygen mask group as reference; 95% confidence intervals (CI) are given in parentheses. Table at the bottom shows the patients at risk per time point

The cumulative proportion of ICU survival, evaluated up to 90 days of ICU admission, differed significantly according to the initially chosen non-invasive oxygenation strategy (p = 0.02) (Fig. 3a). As compared to patients treated with an oxygen mask, the hazard ratio for ICU mortality was 0.75 [95% CI 0.58–0.98] for HFT ventilated patients, and 1.21 [95% CI 0.8–1.83] for NIV treated patients.

In intubated and mechanically ventilated patients, the 90-day ICU mortality did not differ (p = 0.2) between groups (Fig. 3b).

### Secondary analyses

The time from ICU admission to intubation showed no influence on static respiratory system compliance, driving airway pressure, or PaO_2_/FiO_2_ ratio as measured shortly after intubation (Additional file 1: Fig. s8, Additional file 1: Fig. s9, Additional file 1: Fig. s10). However, it was associated with an elevated ICU risk of mortality in a time-varying Cox regression model (Additional file 1: Fig. s11).

The Cox proportional hazards model, in covariate balanced population analysis, showed that highly predictive variables for ICU mortality were age, body mass index, high levels of C-reactive protein, procalcitonin, ferritin, D-dimers, and the presence of chronic obstructive pulmonary disease (Additional file 1: Fig. s12). Variables that were highly predictive of ICU mortality in patients, who required invasive mechanical ventilation, are shown in Additional file 1: Fig. s13. In mechanically ventilated patients, high D-dimers were significantly associated with mortality independently of the static respiratory system compliance (Additional file 1: Fig. s14).

Sensitivity analyses assessing 90-day ICU mortality risk in the un-balanced population in crude models (Additional file 1: Fig. s15, Additional file 1: Table s5) and multivariable-adjusted models (Additional file 1: Fig. s16, Additional file 1: Fig. s17, Additional file 1: Fig. s18, Additional file 1: Fig. s19), with and without between-centre random effects term, showed the same associations as the covariate balanced population analysis.

## Discussion

In this retrospective, baseline covariate balanced, multicentre cohort of 1093 critically ill non-invasively ventilated COVID-19 ICU patients, 897 (82%) eventually required endotracheal intubation and 310 (28%) died during their ICU stay. The need for endotracheal intubation and invasive mechanical ventilation was significantly lower in patients supported by HFT and NIV than in those receiving oxygen mask therapy at ICU admission. Additionally, patients supported by means of HFT had a lower risk of ICU mortality than patients supported with oxygen masks and NIV.

Debate around the optimal choice of non-invasive oxygenation support in acute hypoxemic respiratory failure has re-emerged within the framework of the COVID-19 pandemic [1, 12, 13, 15, 21–23]. The association between the use of HFT or NIV and a reduction in endotracheal intubation rates compared to oxygen mask has yielded ambiguous results [24–26]. The two largest randomized trials to date did not find reductions in endotracheal intubation rates benefitted patients supported with HFT or NIV compared to those treated with an oxygen mask [27, 28] These findings may have several explanations: different populations and etiologies of acute hypoxemic respiratory failure, different inclusion and exclusion criteria, non-identical methodological approaches to the use of non-invasive techniques, and different criteria to declare a failed non-invasive oxygenation attempt. Furthermore, the primary outcomes of these studies were not the same.

In SARS-CoV-2-induced hypoxemic respiratory failure, however, the use of HFT and NIV was superior to oxygen mask in limiting progression to invasive mechanical ventilation, supporting our findings [29, 30]. The reason for this efficacy of HFT and NIV is possibly explained by their physiological effects in terms of improvements in gas exchange, decreases in respiratory muscle effort, and a generally ameliorated sensation of dyspnea as compared to oxygen masks [31–37]. Several mechanisms may explain these beneficial mechanisms. First, the increase in end-expiratory lung volume with some degree of alveolar recruitment induced by PEEP could decrease shunt and consequently improve PaO_2_ [32–34]. Second, as compared to facial oxygen masks, HFT generates a dead space washout of the upper airways, allowing a more homogeneous distribution of tidal volume [32]. Third, HFT induces a decrease in respiratory rate with less effort per breath with unchanged tidal volumes, thus suggesting better lung compliance [32]. These effects may help decrease inspiratory muscle effort, reduce the chemical drive to breath and, in turn, decrease the sensation of dyspnea [35]. It is of note, however, that we are dealing with a single disease (COVID-19) that is typically vasculopathic and accompanied by widespread endothelialitis [38, 39]. Such physiopathological derangements may profoundly modify the ventilation/perfusion relationships and hypoxic pulmonary vasoconstriction [40]. In addition, SARS-CoV-2 exerts direct effects on the carotid bodies and the central nervous system [39], which may alter the central respiratory drive [21].

It is also of interest in our study that we observed lower endotracheal intubation rates in HFT and NIV treated patients than in oxygen mask treated patients. However, only the use of HFT was associated with a reduction in mortality rate. This result is consistent with the landmark trial of Frat et al. [27] but in contrast with the remaining literature [28–30]. Recent data [41] indicate that when compared to early intubation, HFT is associated with an increase in ventilator-free days and no differences in hospital mortality. The lower mortality in the HFT group in our study could directly reflect the lower intubation rate in this population.

In our study, we observed a trend towards higher mortality in the NIV group than in the oxygen mask group despite the lower intubation rate. It has been hypothesized that early endotracheal intubation and lung-protective ventilation in patients failing non-invasive oxygenation support and eventually requiring invasive mechanical intubation is associated with a protective effect. In a large series of ADRS patients, Kangelaris et al.[42] showed that mortality in those who were intubated early (on day 1 of ARDS diagnosis) was significantly lower than in those intubated later (between 2 and 4 days after ARDS diagnosis): the adjusted risk of death was 2.37 times higher for late intubation than for early intubation. Other authors, however, did not observe these effects in COVID-19 patients [43, 44]. More than time per se, several authors have suggested that what occurs during non-invasive oxygenation support is relevant. Carteaux [45] and Frat [46] showed that rather than the time to intubation, large tidal volumes—and thus large transpulmonary pressure swings—during NIV are associated with worse outcomes. Hence, one could argue that the total dose of injury (i.e. size of tidal volume and dissipated pressure times duration of ventilation) may eventually be harmful.

Our data show a clear association between the time from ICU admission to intubation and an increased risk of mortality (see Additional file 1: Fig. s11), possibly explaining the trend towards higher mortality in intubated patients previously undergoing NIV support. Additionally, patient self-inflicted lung injury, through the generation of large swings in transpulmonary pressures with excessive tidal volumes and cycling opening and alveolar collapse phenomena, has been postulated as a primary driver of mortality in NIV supported patients [45–47]. In addition, other relevant phenomena occurring in non-intubated spontaneously breathing subjects with acute hypoxemic respiratory failure include expiratory airway closure [48], *pendelluft* with regionally large swings of transpulmonary pressure [49] and, possibly bronchiolotrauma [50]. Finally, the large transpulmonary pressure swings during spontaneous breathing, which generate high pulmonary transmural vascular pressures, also enhance pulmonary oedema [47, 51]. However, in our study, shortly after endotracheal intubation and irrespectively of the non-invasive oxygenation strategy used, we found that respiratory mechanics and gas exchange were comparable between groups. This observation suggests that, when used for a relatively short period of time, none of these three very different non-invasive oxygenation techniques seem to be worse than the other in terms of magnifying the amount of lung injury (as clinically assessed in terms of gas exchange and basic respiratory system mechanics).

Whether the development of a full-blown patient self-inflicted lung injury requires a time-course threshold, as suggested by the increased mortality in patients intubated after more than 3 days under non-invasive respiratory support (Additional file 1: Fig. s11), or it is governed by another form of time-to-event relationship, is unknown [47]. Conversely, patient self-inflicted lung injury could primarily be biotraumatic, exacerbating pulmonary inflammation. And only secondarily, at a later stage (i.e. invasive mechanical ventilation acting as a “second hit”*),* would this lead to diffuse alveolar damage and alveolar remodelling, and thus impair respiratory system mechanics [30, 47, 49, 52].

Our results stand in contrast with those from a recent randomized controlled trial that compared HFT and NIV as treatment for hypoxemic respiratory failure. The authors found that mortality rates were comparable in the two groups [53]. Nevertheless, it is of note that the patients in Grieco’s study were treated by means of a helmet rather than a facemask. Recent evidence suggests that the limitations of NIV in oxygenation support of hypoxemic respiratory failure are inherent to the facemask interface and that the use of a helmet is associated with fewer air leaks, higher and more stable PEEP levels during prolonged periods of time, improved oxygenation, and reduced inspiratory efforts [31, 54]. Our study has a number of limitations. First, the cohort of patients was collected retrospectively and lacked any degree of randomization, formally impeding an unbiased treatment effect. Nonetheless, the baseline covariate balancing performed enabled mitigation of most objectively assessable confounders. Second, the cohort may be subject to selection bias as during the pandemic outbreak, patients who were treated with the same non-invasive oxygenation techniques but were not admitted into the ICU were not included in the study. Third, in the absence of a study protocol, the choice of oxygenation support strategy and the decision to proceed towards endotracheal intubation and mechanical ventilation were likely variable. It is not known whether the choice of one technique or another was based on clinical decision or equipment availability. Nonetheless, the data presented faithfully represent usual care practice during the pandemic. Fourth, no data on longitudinal inflammatory parameters or pulmonary mechanics data were available, preventing investigation of long-term mechanistic effects associated with the choice of non-invasive oxygenation support strategy. Fifth, neither gas exchange nor other respiratory variables were collected before invasive mechanical ventilation was started. Sixth*,* mortality was available from the ICU only, impeding analysis of hospital or absolute survival. Nevertheless, the largest proportion of mortality in critically ill patients occurs in the ICU, and our patients were followed for up to 90 days therein. Seventh, no specifications were available regarding the type of NIV interface and settings employed, albeit most probably only facemasks were used. Eighth, the temporal gap between ICU admission and endotracheal intubation was only available on a scale of days, thus impeding a more granular analysis. Ninth, the imbalance in group sizes could have led to a lack of power to recognize higher mortality in the NIV supported group. Tenth, as we do not have data from a more contemporaneous cohort, we cannot assess the dynamic effects of the pandemic—such as changing care practices and new pharmacologic treatments—on the reported effects. Finally, the potential role of unmeasured confounders cannot be excluded.

## Conclusion

In critically ill COVID-19 ICU patients with acute hypoxemic respiratory failure, the use of HFT was associated with lower intubation and ICU mortality rates than those in patients supported by means of oxygen mask and NIV. Given the inconclusive data in the ICU population, the choice of HFT as a primary non-invasive oxygenation support strategy seems warranted.

## Supplementary Information

**Additional file 1: e-Table 1:** Percentage of missing values. **e-Appendix 1:** Multiple imputations of missing data and technique break down. **e-Figure 1:** Multiple imputation - convergence plots. **e-Figure 2:** Multiple imputation - distribution plots. **e-Table 2:** Imputation model fit. **e-Appendix 2:** Extended Statistical Methodology. **e-Figure 3:** Covariate balancing models - model fit comparison. **e-Figure 4:** Covariate balancing models - weights comparison. **e-Appendix 3:** Study information. **e-Table 3: **Overall un-balanced population. **e-Table 4:** Un-balanced study population. **e-Figure 5:** Final covariance balancing model - model fit. **e-Figure 6:** Final covariate balancing model - individual variable distributions. **e-Figure 7:** Final covariate balancing model - model weights. **e-Figure 8:** Static compliance versus time from ICU admission until intubation. **e-Figure 9:** Driving pressure versus time from ICU admission until intubation. **e-Figure 10:** PaO_2_/ FiO_2_ ratio versus time from ICU admission until intubation. **e-Figure 11:** Cox time varying model for influence of time until intubation on ICU mortality. **e-Figure 12:** Multivariable Cox proportional hazards model for in-ICU mortality (overall and covariance balanced). **e-Figure 13:** Multivariable Cox proportional hazards model for in-ICU mortality (only intubated and covariance balanced). **e-Figure 14:** Kaplan-Meier curve for ICU mortality – compliance vs. D-dimers. **e-Figure 15:** Kaplan-Meier curve for ICU mortality in unbalanced population. e-Table 5: Cox mixed-effects model with between-center random effects term (unbalanced). **e-Figure 16:** Multivariable Cox proportional hazards model for in-ICU mortality (overall and unbalanced). **e-Figure 17:** Multivariable Cox proportional hazards model for in-ICU mortality (only intubated and unbalanced). **e-Figure 18:** Multivariable Cox mixed-effects model with between-center random effects term for in-ICU mortality (overall study population – unbalanced). **e-Figure 19:** Multivariable Cox mixed-effects model with between-center random effects term for in-ICU mortality (only intubated – unbalanced). **e-Appendix 4:** Formulas.

## Data Availability

The dataset of the current study is available from the corresponding author on reasonable request.

## Abbreviations

COVID-19: Coronavirus disease-2019
ICU: Intensive care unit
SARS-CoV-2: Severe acute respiratory syndrome coronavirus 2
SpO2: Pulse oximeter oxygen saturation
CI: Confidence interval
ARDS: Acute respiratory distress syndrome
PEEP: Positive end-expiratory pressure
Oxygen mask: Standard non-rebreather oxygen masks
HFT: High-flow oxygen therapy administered through large-bore nasal cannulas
NIV: Non-invasive positive pressure ventilation
CPAP: Continuous positive airway pressure
