# Supplementary material for: Non-invasive oxygenation support in acutely hypoxemic COVID-19 patients admitted to the ICU: a multicenter observational retrospective study
Source: Crit Care. 2022 Feb 8;26:37. doi: 10.1186/s13054-022-03905-5 (PMC8822661; doi:10.1186/s13054-022-03905-5)
Supplement: Supplementary file 1 — Additional file 1: e-Table 1: Percentage of missing values. e-Appendix 1: Multiple imputations of missing data and technique break down. e-Figure 1: Multiple imputation - convergence plots. e-Figure 2: Multiple imputation - distribution plots. e-Table 2: Imputation model fit. e-Appendix 2: Extended Statistical Methodology. e-Figure 3: Covariate balancing models - model fit comparison. e-Figure 4: Covariate balancing models - weights comparison. e-Appendix 3: Study information. e-Table 3: Overall un-balanced population. e-Table 4: Un-balanced study population. e-Figure 5: Final covariance balancing model - model fit. e-Figure 6: Final covariate balancing model - individual variable distributions. e-Figure 7: Final covariate balancing model - model weights. e-Figure 8: Static compliance versus time from ICU admission until intubation. e-Figure 9: Driving pressure versus time from ICU admission until intubation. e-Figure 10: PaO2/ FiO2 ratio versus time from ICU admission until intubation. e-Figure 11: Cox time varying model for influence of time until intubation on ICU mortality. e-Figure 12: Multivariable Cox proportional hazards model for in-ICU mortality (overall and covariance balanced). e-Figure 13: Multivariable Cox proportional hazards model for in-ICU mortality (only intubated and covariance balanced). e-Figure 14: Kaplan-Meier curve for ICU mortality – compliance vs. D-dimers. e-Figure 15: Kaplan-Meier curve for ICU mortality in unbalanced population. e-Table 5: Cox mixed-effects model with between-center random effects term (unbalanced). e-Figure 16: Multivariable Cox proportional hazards model for in-ICU mortality (overall and unbalanced). e-Figure 17: Multivariable Cox proportional hazards model for in-ICU mortality (only intubated and unbalanced). e-Figure 18: Multivariable Cox mixed-effects model with between-center random effects term for in-ICU mortality (overall study population – unbalanced). e-Figure 19: Multivariable Cox mixed-effects model with be [file 13054_2022_3905_MOESM1_ESM.pdf]

Supplemental online information

Non-invasive oxygenation support in acutely hypoxemic COVID-19 patients admitted to the ICU: A multicenter observational retrospective study

Wendel-Garcia, Mas, González-Isern, Ferrer, Máñez, Masclans, Sandoval, Vera, Trenado, Fernández, Sirvent, Martínez, Ibarz, Garro, Lopera, Bodí, Yébenes-Reyes, Triginer, Vallverdú, Baró, Bodí, Saludes, Valencia, Roche-Campo, Huerta, Cambra, Barberà, Echevarria, Peñuelas and Mancebo

for the UCIsCAT study group

e-Table 1: Percentage of missing values **4**

e-Appendix 1: Multiple imputations of missing data and technique break down **5**

e-Figure 1: Multiple imputation - convergence plots **6**

e-Figure 2: Multiple imputation - distribution plots **10**

e-Table 2: Imputation model fit **12**

e-Appendix 2: Extended Statistical Methodology **13**

e-Figure 3: Covariate balancing models - model fit comparison **17**

e-Figure 4: Covariate balancing models - weights comparison **18**

e-Appendix 3: Study information **19**

e-Table 3: Overall un-balanced population **21**

e-Table 4: Un-balanced study population **22**

e-Figure 5: Final covariance balancing model - model fit **23**

e-Figure 6: Final covariate balancing model - individual variable distributions **26**

e-Figure 7: Final covariate balancing model - model weights **29**

e-Figure 8: Static compliance versus time from ICU admission until intubation **30**

e-Figure 9: Driving pressure versus time from ICU admission until intubation **31**

e-Figure 10: PaO_2/ FiO_2 ratio versus time from ICU admission until intubation **32**

e-Figure 11: Cox time varying model for influence of time until intubation on ICU mortality **33**

e-Figure 12: Multivariable Cox proportional hazards model for in-ICU mortality *(overall and covariance balanced)* **34**

e-Figure 13: Multivariable Cox proportional hazards model for in-ICU mortality *(only intubated and covariance balanced)* **35**

e-Figure 14: Kaplan-Meier curve for ICU mortality – compliance vs. D-dimers **36**

e-Figure 15: Kaplan-Meier curve for ICU mortality in unbalanced population **37**

e-Table 5: Cox mixed-effects model with between-center random effects term (*unbalanced*) **38**

e-Figure 16: Multivariable Cox proportional hazards model for in-ICU mortality *(overall and unbalanced)* **39**

e-Figure 17: Multivariable Cox proportional hazards model for in-ICU mortality *(only intubated and unbalanced)* **40**

e-Figure 18: Multivariable Cox mixed-effects model with between-center random effects term for in-ICU mortality *(overall study population – unbalanced)* **41**

e-Figure 19: Multivariable Cox mixed-effects model with between-center random effects term for in-ICU mortality *(only intubated – unbalanced)* **42**

e-Appendix 4: Formulas **43**

References **44**

e-Table 1: Percentage of missing values

**Interleukin-6**	79.5%
**Ferritin**	39.9%
**PCT**	31.4%
**Height**	29.3%
**Plateau pressure**	29.3%
**Lactate**	23.0%
**D-dimers**	22.7%
**Weight**	16.7%
**Tidal volume**	9.7%
**Respiratory rate**	5.7%
**$PaCO_2$**	3.7%
**CRP**	3.5%
**pH**	3.3%
**PaO_2**	3.0%
**PEEP**	2.4%
**Lymphocytes**	2.2%
**FiO_2**	0.9%
**Leucocytes**	0.4%

List of abbreviations: CRP – C-reactive protein; PCT – procalcitonin; PEEP - positive end-expiratory pressure; $PaCO_2$ - partial pressure of arterial carbon dioxide; PaO_2 - partial pressure of arterial oxygen; FiO_2 - inspiratory fraction of oxygen

e-Appendix 1: Multiple imputations of missing data and technique break down

The missing at random assumption was postulated after assessing for quasi-missingness at random through consideration of survival curves, the log-rank test and Cox proportional hazards models, evaluating the interaction between variables presenting the highest missing rates and a possible pattern on diverging mortality rates, which was not present [1]. Additionally, delta-adjustment sensitivity analyses were performed, which indicated no departure from the missing at random assumption [2]. All independent baseline variables recorded in the data set were included. For each variable, a linear regression model accounting for all non-collinear and non-intercepting variables was specified. We then used a multiple imputation missingness pattern approach as covariate in each linear regression model to account for potential information intrinsically present in missingness patterns [3]. Five parallel imputation models with 100 iterations each were run.

Whilst it has long been postulated that a cut-off of about 40% should be used to remove variables from analyses[4], recent research is increasingly showing that missingness percentage itself shouldn't govern this choice and that cut-offs are mostly arbitrary [5-7]. In studies with a large number of patients (>500-1000), containing many variables that can be regarded as at least partially explanatory for other covariates, multiple imputation, especially by means of predictive mean matching, might offer estimates very close to "reality" [5, 7]. It has been shown that the bias of a variables imputation is mainly governed, not by its percentage missingness, but by the amount of other covariates included to the model, which might predict or correlate with the missing parameter [5]. Indeed multiple imputation was designed in order for compensate high percentages of missingness up to 70-80% [2]. Inclusion of missingness patterns methods to the imputation model, might even improve estimates [8, 9].

Having proposed the multiple imputation equations, in our case including all baseline characteristics, center clustering factors, temporality as well as missingness patterns, a good diagnostic tool to assess the plausibility of the imputation is the convergence of imputation plots (e-Figure 1). As can be observed means and standard deviations of the imputed variables have little to no variation during consecutive imputation cycles and the five models present similar values, suggesting imputation model robustness. Additionally, when comparing the distributions of the imputed missing variables and those of the original distributions, standardized means are below 0.1 and variance ratios below 2, suggesting an excellent overlap of original and imputed distributions (e-Figure 2 and e-Table 2).

e-Figure 1: Multiple imputation - convergence plots

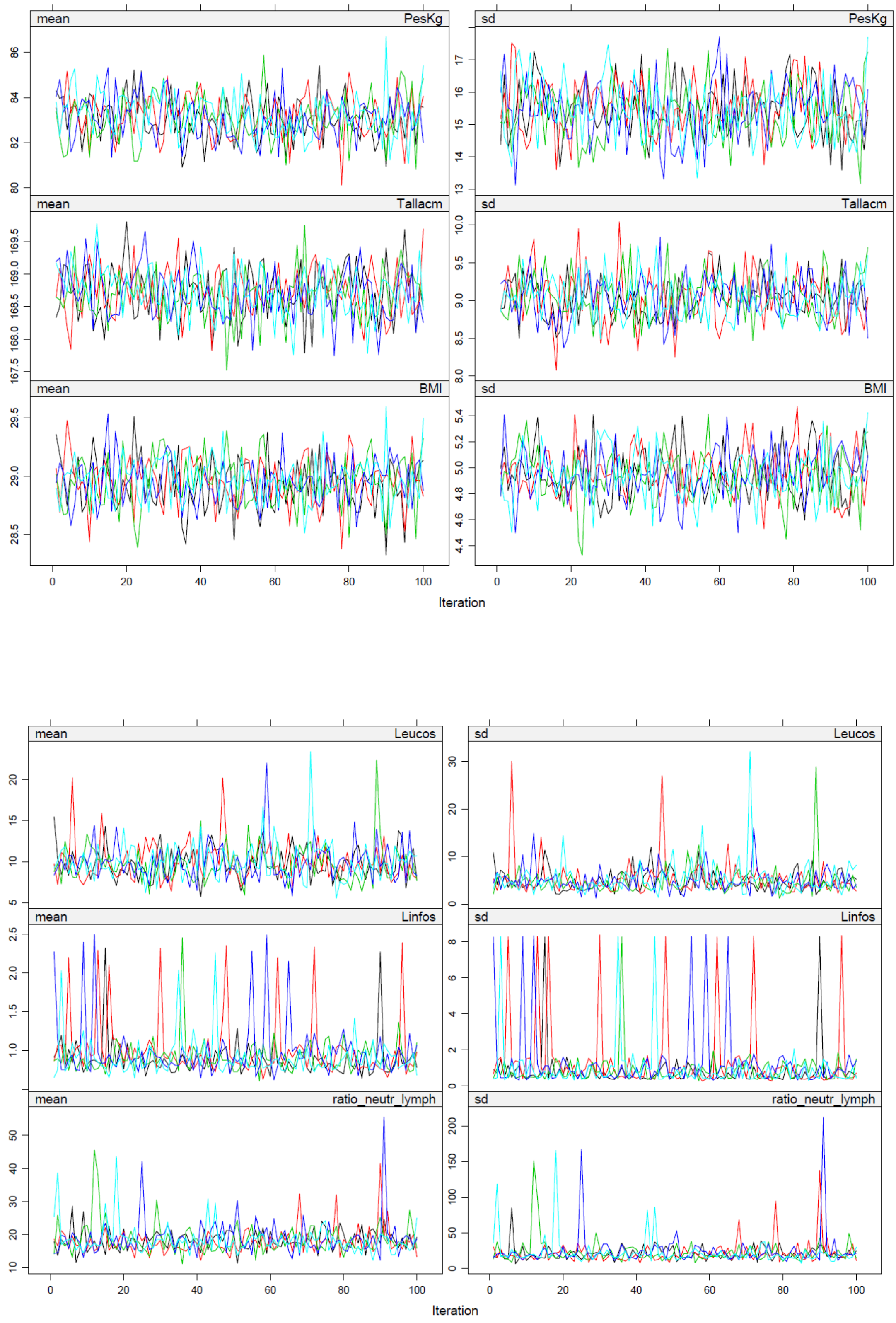

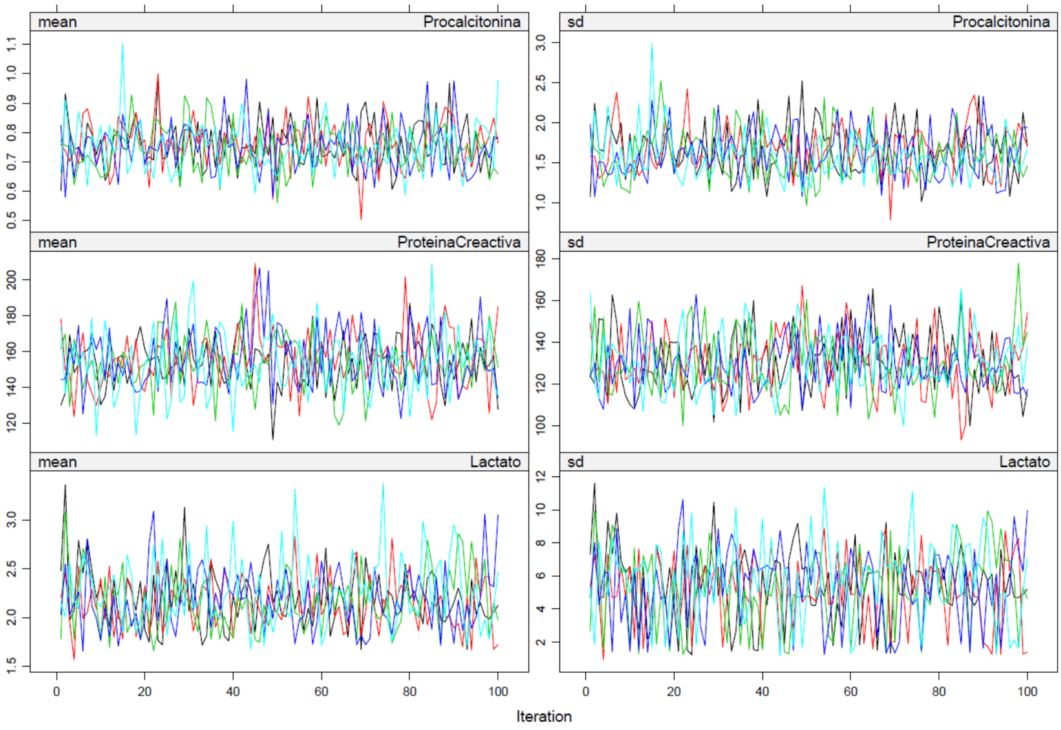

mean
Procalcitonina
sd
Procalcitonina
mean
ProteinaCreactiva
sd
ProteinaCreactiva
mean
Lactato
sd
Lactato
Iteration

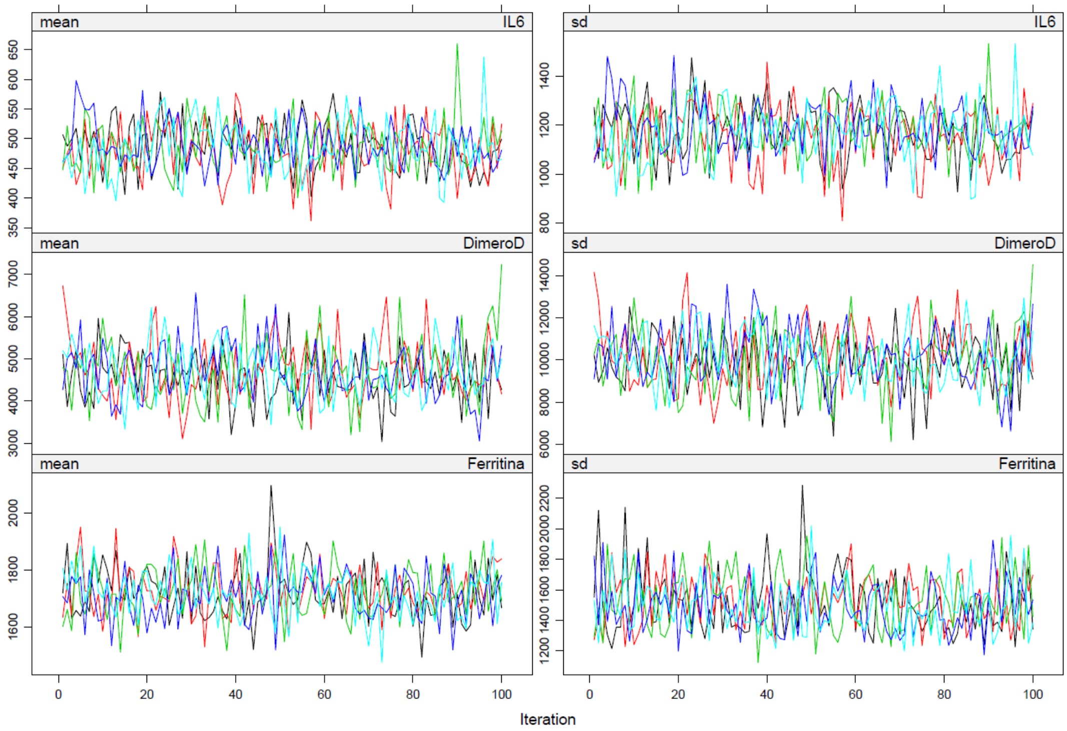

mean
IL6
sd
IL6
mean
DimeroD
sd
DimeroD
mean
Ferritina
sd
Ferritina
Iteration

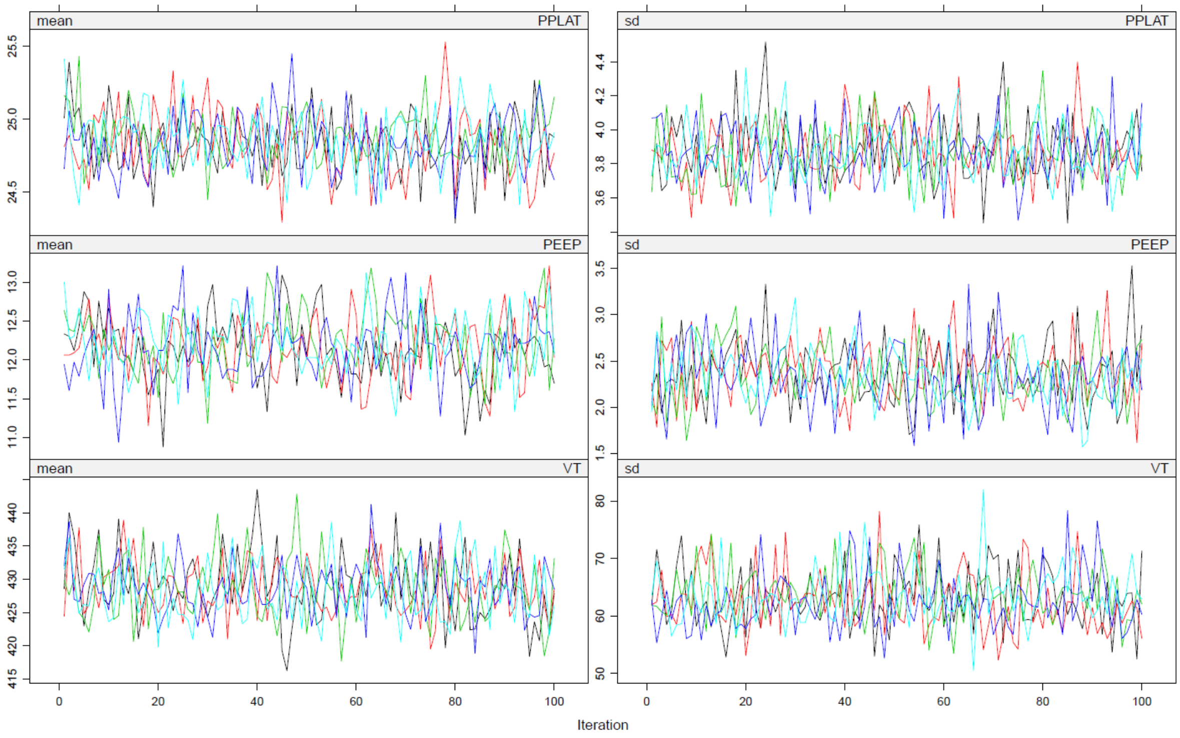
mean
PPLAT
sd
PPLAT
mean
PEEP
sd
PEEP
mean
VT
sd
VT
Iteration

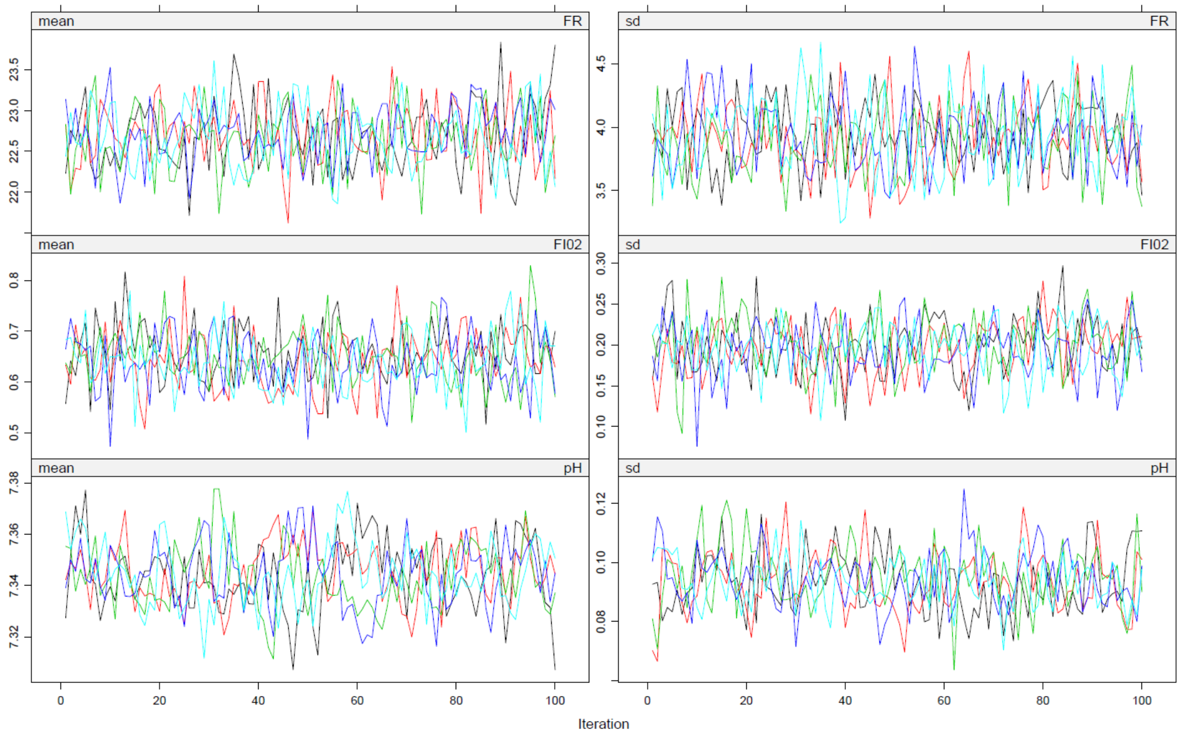
mean
FR
sd
FR
mean
FI02
sd
FI02
mean
pH
sd
pH
Iteration

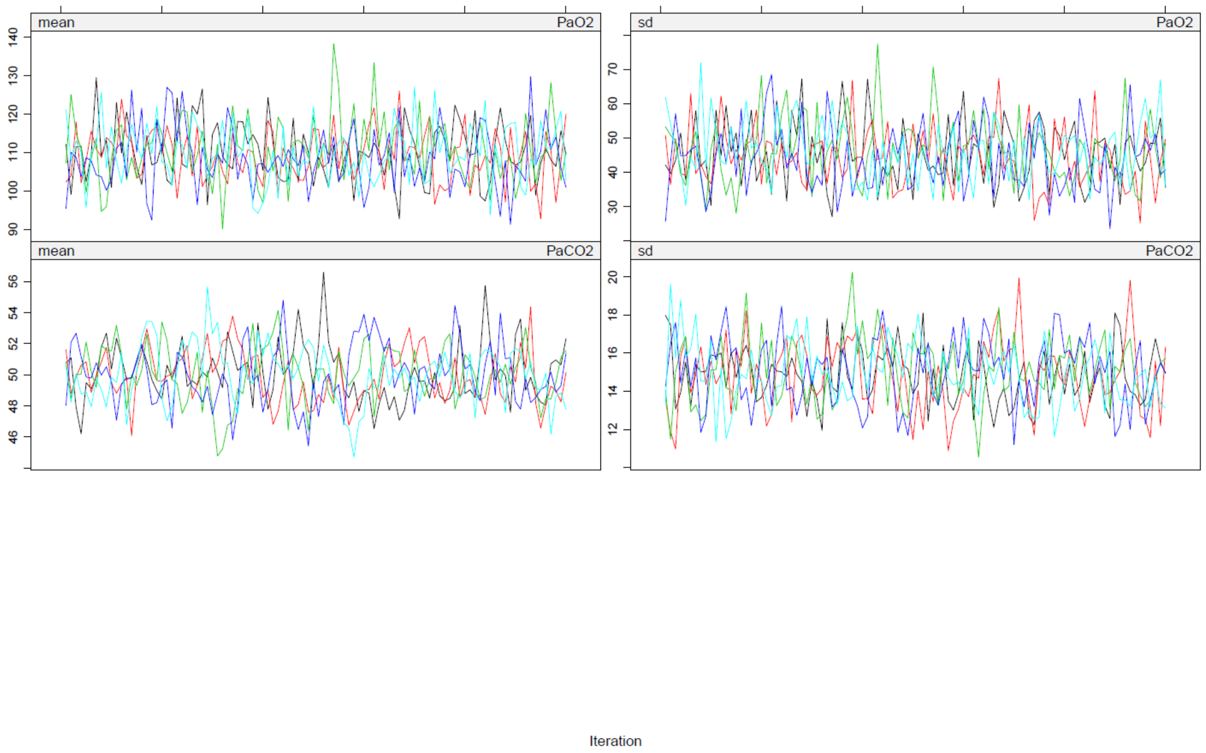

Change in mean and standard deviation per imputation iteration cycle for all imputed variables and imputation models (5 models). List of abbreviations: PesKG - weight; Tallacm - height; BMI – body mass index; Leucos - leucocytes; Linfos - lymphocytes; ratio_neutr_lymph – neutrophil/ lymphocyte ratio; Procalcitonina – procalcitonin; ProteinaCreactiva – C-reactive protein; Lactato – lactate; IL6 – interleukin-6; DimeroD – D-dimers; Ferritina – ferritin; PPLAT – Plateau pressure; PEEP - positive end-expiratory pressure; VT – tidal volume; FR – respiratory rate; FiO_2 - inspiratory fraction of oxygen; PaO_2 - partial pressure of arterial oxygen; $PaCO_2$ - partial pressure of arterial carbon dioxide

e-Figure 2: Multiple imputation – distribution plots

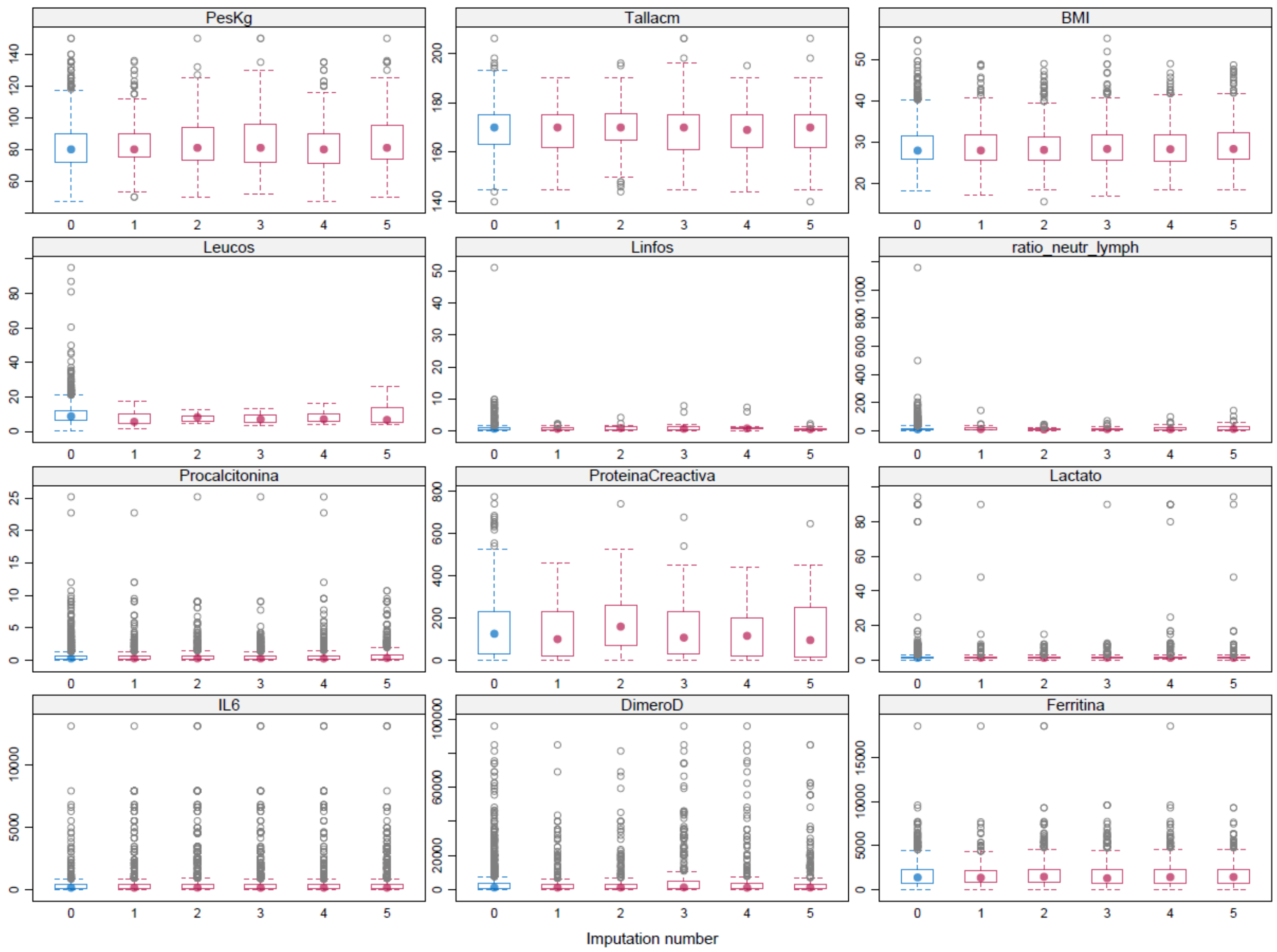

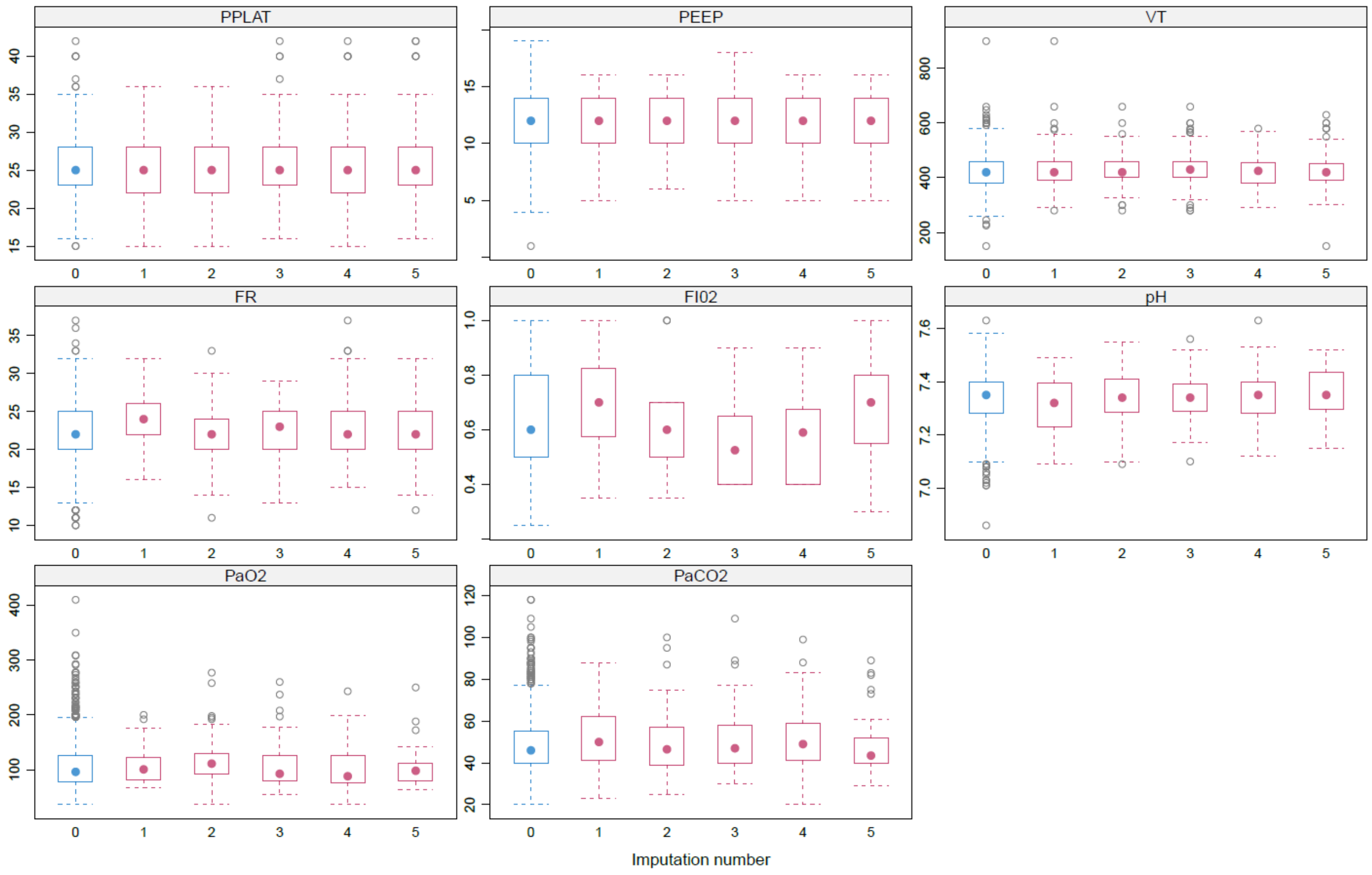

Distribution box-plots for imputed variables. The distributions of the variables post imputation (5 models) are plotted in red, as opposed to the original distribution which is plotted in blue. List of abbreviations: PesKG - weight; Tallacm - height; BMI – body mass index; Leucos - leucocytes; Linfos - lymphocytes; ratio_neutr_lymph – neutrophil/ lymphocyte ratio; Procalcitonina – procalcitonin; ProteinaCreactiva – C-reactive protein; Lactato – lactate; IL6 – interleukin-6; DimeroD – D-dimers; Ferritina – ferritin; PPLAT – plateau pressure; PEEP - positive end-expiratory pressure; VT – tidal volume; FR – respiratory rate; FiO_2 - inspiratory fraction of oxygen; paO_2 - partial pressure of arterial oxygen; $paCO_2$ - partial pressure of arterial carbon dioxide

e-Table 2: Imputation model fit

	Original	Model 1	p	SMD	VR	Model 2	p	SMD	VR	Model 3	p	SMD	VR	Model 4	p	SMD	VR	Model 5	p	SMD	VR
n	1703	1703				1703				1703				1703				1703			
**Weight**	82.7 (15.4)	82.8 (15.4)	0.792	0.009	1.003646	82.9 (15.4)	0.679	0.015	0.997363	83.1 (15.7)	0.519	0.023	1.093468	82.6 (15.5)	0.834	0.008	1.031941	83.2 (15.8)	0.417	0.029	1.120704
**Height**	168.9 (9.1)	168.8 (9.1)	0.738	0.013	0.990133	169.1 (9.1)	0.487	0.026	0.992783	168.8 (9.3)	0.801	0.010	1.078529	168.7 (8.9)	0.587	0.020	0.926292	168.8 (9.3)	0.709	0.014	1.063739
**BMI**	29.1 (5.2)	29.1 (5.1)	0.955	0.002	0.982469	29.0 (5.1)	0.673	0.016	0.961262	29.2 (5.2)	0.730	0.013	1.029542	29.0 (5.1)	0.730	0.013	0.98666	29.2 (5.2)	0.547	0.023	1.067739
**Leucocytes**	10.0 (6.5)	10.0 (6.5)	0.966	0.001	0.997402	10.0 (6.5)	0.969	0.001	0.993887	10.0 (6.5)	0.965	0.001	0.994975	10.0 (6.5)	0.975	0.001	0.995191	10.1 (6.5)	0.990	<0.001	1.003075
**Lymphocytes**	0.9 (1.6)	0.9 (1.5)	0.952	0.002	0.962468	0.9 (1.5)	0.985	0.001	0.965922	0.9 (1.6)	0.945	0.002	0.995854	0.9 (1.6)	0.956	0.002	0.991536	0.9 (1.5)	0.919	0.004	0.961449
**Neutrophils/ lymphocytes**	17.3 (36.4)	17.3 (36.2)	0.974	0.001	0.975866	17.2 (36.1)	0.945	0.002	0.961428	17.3 (36.1)	0.980	0.001	0.96391	17.3 (36.2)	0.981	0.001	0.971662	17.5 (36.3)	0.893	0.005	0.986905
**PCT**	0.7 (1.6)	0.8 (1.7)	0.831	0.008	1.060655	0.7 (1.7)	0.894	0.005	1.068059	0.7 (1.6)	0.684	0.015	0.881047	0.8 (1.7)	0.829	0.008	1.282374	0.8 (1.6)	0.229	0.046	1.048801
**CRP**	147.6 (128.7)	146.9 (128.3)	0.875	0.005	0.98874	149.0 (129.8)	0.769	0.010	1.034986	147.8 (129.3)	0.977	0.001	1.017702	147.2 (128.2)	0.917	0.004	0.984755	147.2 (129.0)	0.924	0.003	1.009864
**Lactate**	2.4 (7.4)	2.3 (6.9)	0.807	0.009	0.783639	2.2 (6.5)	0.537	0.023	0.608241	2.3 (6.8)	0.704	0.014	0.741842	2.6 (8.0)	0.601	0.019	1.420788	2.4 (7.3)	0.958	0.002	0.967323
**Interleukine-6**	459.2 (1134.4)	489.1 (1220.7)	0.646	0.025	1.340824	508.4 (1252.6)	0.460	0.041	1.486408	499.8 (1229.6)	0.536	0.034	1.380232	475.3 (1241.6)	0.807	0.014	1.434998	463.6 (1091.3)	0.941	0.004	0.856372
**D-dimers**	4745.2 (10256.7)	4637.3 (10002.0)	0.771	0.011	0.904305	4614.4 (10077.6)	0.725	0.013	0.931948	5309.2 (11414.8)	0.160	0.052	1.534056	4873.8 (10847.8)	0.741	0.012	1.25119	4801.1 (10517.5)	0.884	0.005	1.105657
**Ferritin**	1736.9 (1448.8)	1709.0 (1407.4)	0.621	0.020	0.890546	1777.5 (1552.6)	0.498	0.027	1.318977	1718.9 (1439.1)	0.753	0.012	0.973493	1754.4 (1483.1)	0.763	0.012	1.0983	1744.5 (1422.1)	0.892	0.005	0.928268
n	1387					1387				1387				1387				1387			
**Plateau pressure**	25.2 (3.6)	25.1 (3.7)	0.508	0.029	1.047581	25.1 (3.7)	0.379	0.038	1.085795	25.2 (3.7)	0.929	0.004	1.082424	25.0 (3.8)	0.216	0.054	1.225912	25.1 (3.8)	0.558	0.026	1.1633
**PEEP**	12.3 (2.4)	12.3 (2.4)	0.877	0.006	1.02459	12.3 (2.4)	0.947	0.003	1.012438	12.3 (2.4)	0.999	<0.001	1.013956	12.3 (2.4)	0.960	0.002	0.991194	12.3 (2.4)	0.954	0.002	1.005014
**Tidal volume**	423.8 (63.1)	424.4 (64.2)	0.837	0.008	1.072991	424.5 (62.2)	0.805	0.010	0.945697	425.1 (63.0)	0.632	0.019	0.995169	424.5 (62.8)	0.810	0.010	0.976941	424.1 (63.1)	0.909	0.005	0.997286
**Respiratory rate**	22.4 (3.9)	22.5 (3.9)	0.448	0.030	0.984072	22.4 (3.9)	0.902	0.005	0.972645	22.4 (3.9)	0.871	0.006	0.958686	22.4 (3.9)	0.740	0.013	1.013293	22.4 (3.9)	0.860	0.007	0.996615
**FiO2**	0.6 (0.2)	0.6 (0.2)	0.943	0.003	0.99983	0.6 (0.2)	0.990	<0.001	1.000808	0.6 (0.2)	0.935	0.003	0.998076	0.6 (0.2)	0.942	0.003	0.995516	0.6 (0.2)	0.971	0.001	1.000238
**pH**	7.3 (0.1)	7.3 (0.1)	0.763	0.012	1.028387	7.3 (0.1)	0.963	0.002	1.005223	7.3 (0.1)	0.983	0.001	0.989413	7.3 (0.1)	0.960	0.002	1.002062	7.3 (0.1)	0.917	0.004	1.000921
**PaO2**	107.3 (43.5)	107.3 (43.3)	0.967	0.002	0.979093	107.7 (43.7)	0.822	0.009	1.021343	107.4 (43.6)	0.962	0.002	1.013212	107.1 (43.4)	0.910	0.004	0.992379	107.1 (43.3)	0.935	0.003	0.980736
**PaCO2**	48.8 (13.7)	48.9 (13.8)	0.803	0.010	1.017694	48.8 (13.8)	0.923	0.004	1.030506	48.9 (13.8)	0.844	0.008	1.025061	48.9 (13.8)	0.854	0.007	1.015161	48.7 (13.7)	0.944	0.003	0.992782

Variable distributions, mean (standard deviation), for all imputed variable. P-values, standardized mean differences (SMD) and variance ratios (VR) assess each imputation fit against the original. List of abbreviations: BMI – body mass index; PCT – procalcitonin; CRP – C reactive protein; PEEP - positive end-expiratory pressure; $paCO_2$ - partial pressure of arterial carbon dioxide; paO_2 - partial pressure of arterial oxygen; FiO_2 - inspiratory fraction of oxygen

e-Appendix 2: Extended Statistical Methodology

We employed the causal inference modelling framework suggested by Rubin and Rosenbaum and included all relevant observed covariates at ICU admission into our covariate balancing models [10, 11]. Following covariate balancing we performed a post-balancing analysis of all other measured baseline variables assessing any possible imbalance, as suggested by Rubin and Stuart [12, 13].

Covariate Variable selection

Age, sex, body mass issndex, the time between hospital and ICU admission, comorbidities (cardiovascular, diabetes, cancer, COPD and immunosuppression), leucocyte counts, the neutrophil-to-lymphocyte ratio, procalcitonin levels, C-reactive protein levels, interleukin-6 levels, D-dimer levels, ferritin levels and arterial lactate levels at the time-point of ICU admission were selected as covariates. These variables were chosen as they allowed an equilibrated assessment of relevant demographic characteristics, degree of organ dysfunction, inflammatory dysregulation as well as microcirculatory impairment [14].

Covariate Balancing Algorithm selection

Nine different covariate-balancing algorithms were tested against each other, namely:

1. **Classic propensity score weighting [15]:** This is the classic method known as inversed probability of treatment weighting (IPTW). Specific patient weights are calculated from the probability of receiving a specific intervention given a set of baseline covariates, which is defined as the propensity score. The propensity score for each patient is estimated by means of logistic regression. The model states that the logit function of the probability of receiving an intervention is given by a linear combination of covariates.
2. **Generalized boosted models [16]:** Generalized boosted models are a combination of decision tree algorithms and boosting methods. These models repeatedly fit multiple decision trees to improve model accuracy. Each new model tree regards the miss-modeled data in the preceding trees, and weights the input data so that it gains more priority in succeeding trees. In this way, a propensity score is sequentially constructed improving its fit in every successive branching. Normally a standardized mean difference or maximum Kolmogorov-Smirnov statistic optimization is targeted.
3. **Covariate balance propensity score [17]:** Covariate balance propensity score employs the duality of propensity scores as a covariate balancing score and the probability of treatment assignment conditioned on the covariates. The moment conditions implied by covariate balancing are combined with a standard estimation procedure, such as empirical likelihood or generalized method of moments, to estimate the propensity score. In this way the resulting parametric propensity score is specified so that covariate balance is maximized.

4. **Nonparametric covariate balancing propensity score weighting [17]:** This method maximizes the empirical likelihood of observing an intervention given the observed covariates, constraining the weights so that covariate balance and the original mean of interventions and covariates are ensured. Thus inverse generalized propensity weights are estimated without the generation of a fully parametrized model for each intervention and covariate.
5. **Entropy balancing [18]:** This method attempts to solve an optimization problem consisting in exactly balancing all covariates over as many moments as defined by the modeler and thus directly defining the weights of each patient directly. The optimization problem is constrained by covariate balance, weight positivity and a total weight sum. This method does not estimate patient weights through a propensity score.
6. **Empirical balancing calibration weighting [19]:** This method removes imbalance in all covariates by directly modifying the missspecified uniform weights without directly employing a propensity score. Calibration weights are constructed from moment balancing conditions generating an exact three-way balance between the interventions, the controls and the joint population.
7. **Targeted stable balancing weights using optimization [20]:** This method finds the weights of minimum variance that balance the empirical distribution of the initial covariates through specification of a pre-defined convex optimization problem. Thus, the variance of the individual weights is balanced against the maximal adjustment constraint of each covariate.
8. **Bayesian additive regression trees weighting [21]:** This method is a non-parametric Bayesian regression approach, which employs dimensionally adaptive random basis elements or a sum-of-trees model. By defining a prior and a likelihood a posterior distribution enabling inference of point and interval estimates of the propensity score function and of the marginal covariate effects.

9. **Energy balancing [22]:** This method directly attempts to balance the weighted covariate distributions through a model-free approach, focusing on the full covariate distributions and not just lower order moments of the same. For this purpose a specific distance metric, the energy distance, is used, which is based on powers of the classic Euclidean distance. This energy distance between interventions and the full population is minimized by setting the weights.

Covariate balancing algorithm evaluation

Covariate balancing quality was evaluated based on the difference in mean or prevalence and the higher-order moments and interactions for each covariate between the intervention and the control group in the matched population. Additionally distribution plots for each covariate were inspected [23].

Difference in mean/ prevalence [24, 25]: Standardized Mean Difference (SMD) = $\frac{|\bar{x}_I - \bar{x}_C|}{\sqrt{\frac{(s_I)^2 + (s_C)^2}{2}}}$ → SMD ≤0.1

Higher-order moments and interactions [12, 25]: Variance Ratio (VR) = $\frac{(s_T)^2}{(s_C)^2}$ → 0.5 ≤ VR ≤ 2.0

e-Figure 3: Covariate balancing models - model fit comparison

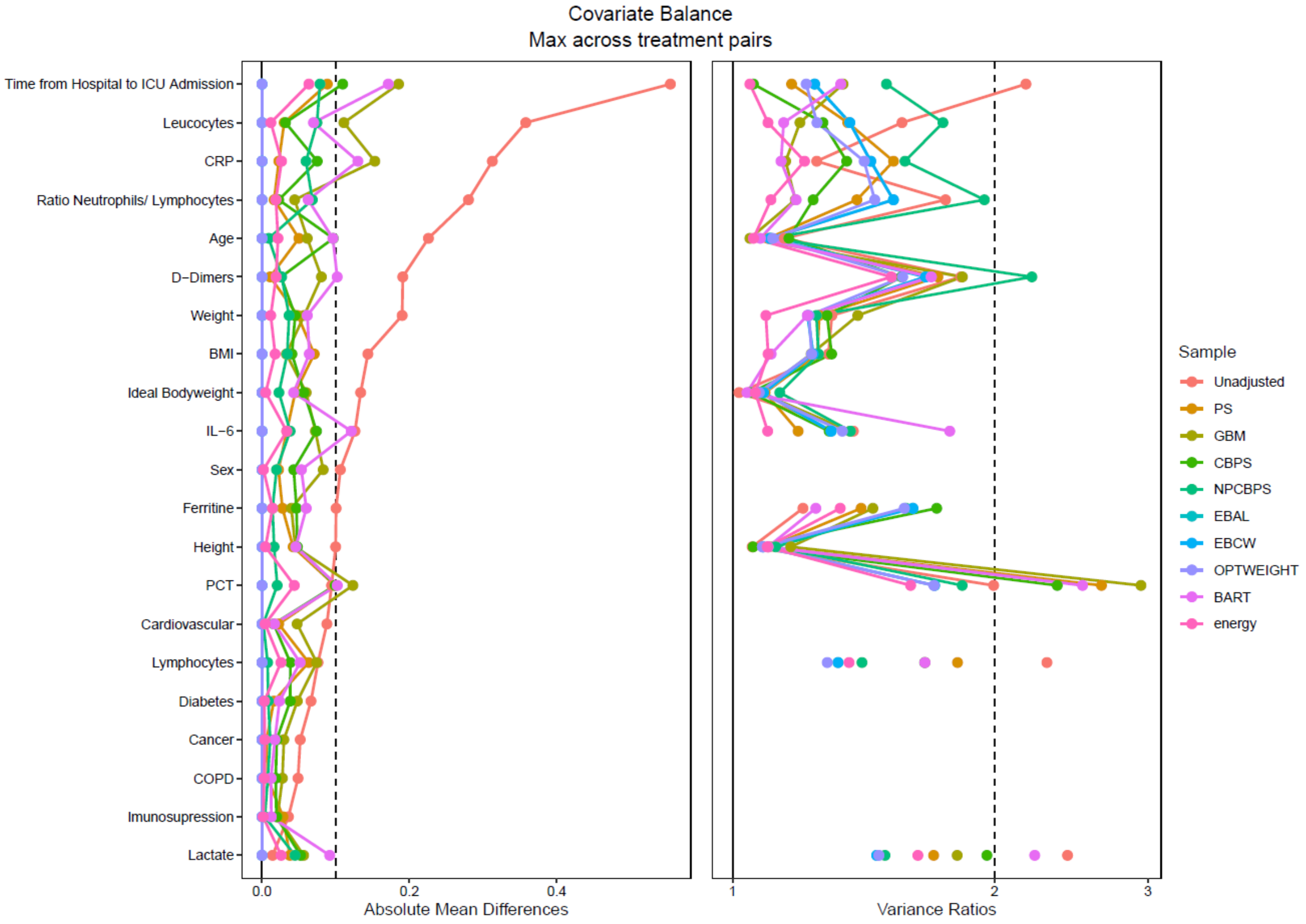

Difference in maximal standardized mean differences (SMD) and variance ratios (VR) between the unbalanced population (in orange) and the nine tested covariance balancing algorithms for each baseline variable. An SMD value <0.1 represents a negligible difference between group means and a VR <2 a negligible difference between higher-order moments and interactions between groups. Algorithms: classic inverse probability weighting (PS), generalized boosted models (GBM), covariate balancing propensity score weighting (CBPS), nonparametric covariate balancing propensity score weighting (NPCBPS), entropy balancing (EBAL), empirical balancing calibration weighting (EBCW), targeted stable balancing weights using optimization (OPTWEIGHT), Bayesian additive regression trees weighting (BART) and energy balancing (ENERGY).

e-Figure 4: Covariate balancing models - weights comparison

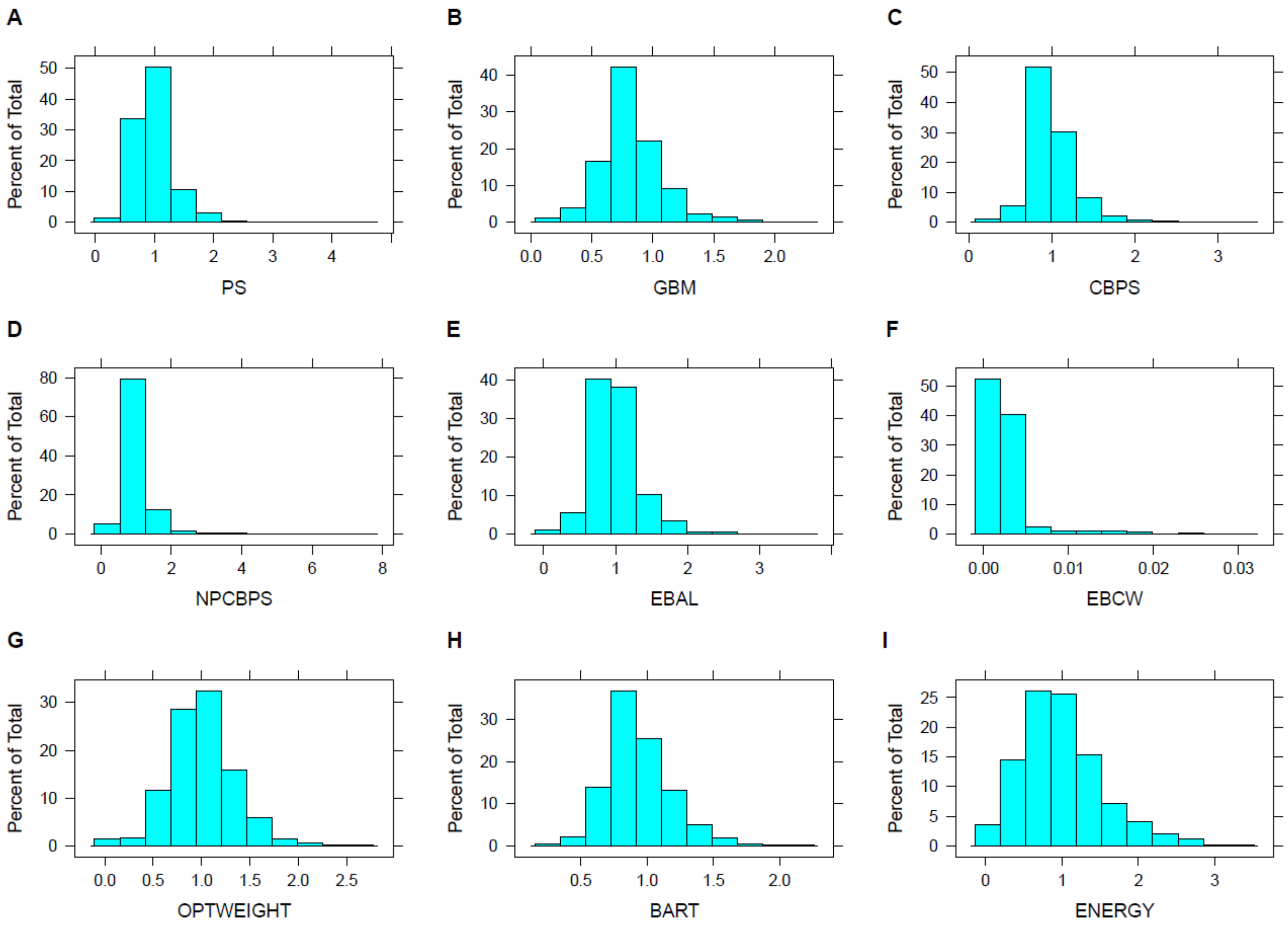

Histograms assessing covariance balancing weight distributions for each tested algorithm. A Gaussian distribution around the 1 without extreme weights is regarded as optimal. Algorithms: classic inverse probability weighting (PS), generalized boosted models (GBM), covariate balancing propensity score weighting (CBPS), nonparametric covariate balancing propensity score weighting (NPCBPS), entropy balancing (EBAL), empirical balancing calibration weighting (EBCW), targeted stable balancing weights using optimization (OPTWEIGHT), Bayesian additive regression trees weighting (BART) and energy balancing (ENERGY).

e-Appendix 3: Study information

UCIsCAT centres and local investigators

- **Hospital Universitari General de la Vall d'Hebron, Barcelona:** R.Ferrer, O.Roca, X. Nuvials, J.C Ruiz, E. Papiol
- **Hospital Universitari de Bellvitge, L'Hospitalet de Llobregat :** R. Mañez, V.D Gumicio
- **Hospital Clínic i Provincial de Barcelona, Barcelona:** E.Sandoval, G.Muñoz, D.Toapanta, P.Castro, J. Osorio
- **Hospital del Mar, Barcelona:** J.R Masclans , R. Muñoz-Bermúdez, F. Parrilla, P. Pérez-Teran, J. Marin-Corral
- **Hospital de Sant Joan Despí Moisès Broggi, Sant Joan Despí:** A. Mas, B. Cancio, S.Hernández-Marín, M.R. Koborzan , C.A Briones
- **Hospital Mútua de Terrassa, Terrassa:** J. Trenado
- **Althaia (Xarxa Assistencial Universitària de Manresa), Manresa:** R.Fernández
- **Hospital Universitari Doctor Josep Trueta de Girona, Girona:** J.M Sirvent, P. Sebastian, X. Saiz
- **Hospital General De Cataluña, Sant Cugat del Vallès:** M. Martínez
- **Hospital Universitari Sagrat Cor - Grup Quirónsalut, Barcelona:** M. Ibarz
- **Hospital General de Granollers, Granollers:** P.Garro, C.Pedrós, E.Vendrell
- **Hospital General de Vic (Consorci Hospitalari de Vic), Vic:** J.L Lopera
- **Hospital Universitari de Tarragona Joan XXIII, Tarragona:** M. Bodí, A. Rodríguez, G. Moreno
- **Hospital de Mataró, Mataró:** J.C Yebenes-Reyes
- **Hospital d'Igualada, Igualada:** C. Triginer
- **Hospital Sant Joan de Reus, Reus:** I. Vallverdú
- **Hospital de Santa Caterina, Salt:** A. Baró , M. Morales
- **Hospital de Sant Pau i Santa Tecla, Tarragona:** F. Bodí
- **Hospital HM Delfos, Barcelona:** P.Saludes, J-R.Cervelló
- **Hospital El Pilar - Grup Quirónsalut, Barcelona:** M. Valencia
- **Hospital de Tortosa Verge de la Cinta, Tortosa:** F.Roche-Campo, D. Franch-Llasat
- **Clínica Sagrada Família, Barcelona:** A.Huerta, P.Santigosa
- **Hospital Sant Joan de Déu, Esplugues de Llobregat:** F.J Cambra, S. Benito
- **Hospital Santa María, Lleida:** C.Barberà
- **Hospital ASEPEYO de Barcelona, Sant Cugat del Vallés:** J.Echevarría
- **Hospital de la Santa Creu i Sant Pau, Barcelona:** J. Mancebo, P.Vera, J-A.Santos, J.Baldirà, A-J.Betbesé, M.Izura, I.Morán, J-C.Suárez, L.Zapata, N.Rodríguez, M.Torrens, A.Cordón, C.Gomila, M.Flores, A.Segarra, M.Morales, L.Mateo, M.Martos, C.González Isern (Coordinating centre)

List of colors: Blue = Catalan public health system hospitals; **Orange** = Catalan private hospitals

UCIsCAT centres, ICU beds and patients

26
Intensive care units

500
ICU beds

Catalan public health system hospitals

120
ICU beds

13
Intensive care units

Catalan private hospitals

Participating public hospitals

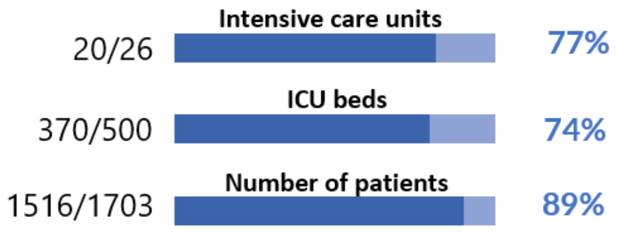

Participating private hospitals

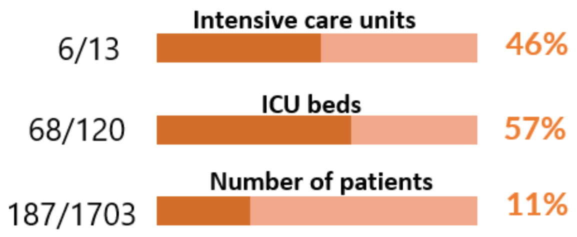

e-Table 3: Overall un-balanced population

	Overall<br>n = 1703
Age (years)	63 [54 - 70]
Female sex	517 (30)
BMI (kg/m²)	28.0 [25.8 - 31.6]
Time from hospital admission to ICU admission [days]	1 [0 - 3]
**Comorbidities**	
Cancer	120 (7)
Diabetes	352 (21)
COPD	119 (7)
Cardiovascular	844 (50)
Immunosuppression	77 (5)
Leucocytes (G/l)	9 [6 - 12]
Lymphocytes (G/l)	0.7 [0.5 - 1.0]
Neutrophils/ lymphocytes	11 [7 - 18]
PCT (µg/l)	0.3 [0.1 - 0.6]
CRP (mg/l)	124 [28 - 231]
Lactate (mmol/l)	1.4 [1.1 - 1.9]
Interleukin-6 (ng/l)	133 [50 - 371]
D-dimers (µg/l)	1160 [553 - 3240]
Ferritin (µg/l)	1387 [787 - 2258]
**Invasive mechanical ventilation before or at ICU admission**	270 (16)
**Non-invasive respiratory support in ICU**	
High-flow oxygen therapy	583 (34)
Non-rebreather oxygen mask	563 (33)
Non-invasive ventilation	256 (15)
Nasal prongs or without respiratory support	31 (2)
Intubation	1387 (81)
Ventilator-free days	12 [0 - 25]
Length of stay ICU (days)	13 [7 - 25]
ICU mortality	504 (30)

Quantitative data are expressed as median [interquartile range] or counts (percentages). BMI – body mass index; COPD – chronic obstructive pulmonary disease; CRP – C-reactive protein; ICU – intensive care unit; PCT – procalcitonin.

e-Table 4: Un-balanced study population

	Oxygen mask<br>n = 553	High-flow oxygen therapy<br>n = 439	Non-invasive ventilation<br>n = 101	p	SMD
Age (years)	64 [55 - 71]	61 [52 - 69]	64 [57 - 70]	0.005	0.151
Female sex	179 (32)	151 (34)	24 (24)	0.120	0.157
BMI (kg/m^2)	27.9 [25.6 - 31.2]	27.9 [26.0 - 31.2]	28.3 [25.8 - 32.7]	0.593	0.095
Time from hospital admission to ICU admission [days]	1.0 [0.0 - 3.0]	2.0 [0.0 - 3.0]	3.0 [1.0 - 5.0]	<0.001	0.360
**Comorbidities**					
Cancer	37 (7)	31 (7)	12 (12)	0.177	0.120
Diabetes	130 (24)	74 (17)	22 (22)	0.035	0.111
COPD	47 (8)	22 (5)	10 (10)	0.060	0.125
Cardiovascular	283 (51)	186 (42)	51 (50)	0.018	0.118
Immunosuppression	23 (4)	34 (8)	6 (6)	0.055	0.102
Leucocytes (G/l)	8.9 [6.3 - 12.3]	7.6 [5.9 - 11.1]	9.7 [7.1 - 13.1]	<0.001	0.240
Lymphocytes (G/l)	0.7 [0.5 - 1.0]	0.8 [0.5 - 1.0]	0.7 [0.5 - 1.0]	0.724	0.053
Neutrophils/ lymphocytes	10.9 [6.7 - 18.1]	9.1 [6.3 - 15.9]	11.5 [7.3 - 21.2]	0.007	0.187
PCT (µg/l)	0.3 [0.1 - 0.7]	0.2 [0.1 - 0.5]	0.2 [0.1 - 0.5]	0.003	0.065
CRP (mg/l)	143 [38 - 236]	79 [12 - 192]	119 [30 - 240]	<0.001	0.210
Lactate (mmol/l)	1.4 [1.0 - 1.9]	1.4 [1.1 - 1.9]	1.5 [1.2 - 2.0]	0.009	0.010
Interleukin-6 (ng/l)	132 [47 - 285]	126[61 - 364]	112 [43 - 326]	0.366	0.085
D-dimers (µg/l)	1301 [630 - 3240]	920 [451 - 2238]	1500 [740 - 5042]	<0.001	0.131
Ferritin (µg/l)	1350 [815 - 2294]	1415 [681 - 2091]	1566 [840 - 2347]	0.370	0.067
Intubation	506 (92)	298 (68)	87 (86)	<0.001	0.410
Length of stay ICU (days)	15.0 [7.0 - 26.0]	12.0 [6.0 - 26.0]	14.0 [7.8 - 24.5]	0.452	0.031
Ventilator-free survival [days]	7.0 [0.0 - 22.0]	18.0 [0.0 - 30.0]	5.0 [0.0 - 21.0]	<0.001	0.261
ICU mortality	176 (32)	94 (21)	41 (41)	<0.001	0.281

Quantitative data are expressed as median [interquartile range]. P-values are given for the difference between respiratory strategies. Standardized mean differences (SMD) define the maximal mean difference between groups. BMI – body mass index; COPD – chronic obstructive pulmonary disease; CRP – C-reactive protein; ICU – intensive care unit; PCT – procalcitonin.

e-Figure 5: Final covariance balancing model – model fit

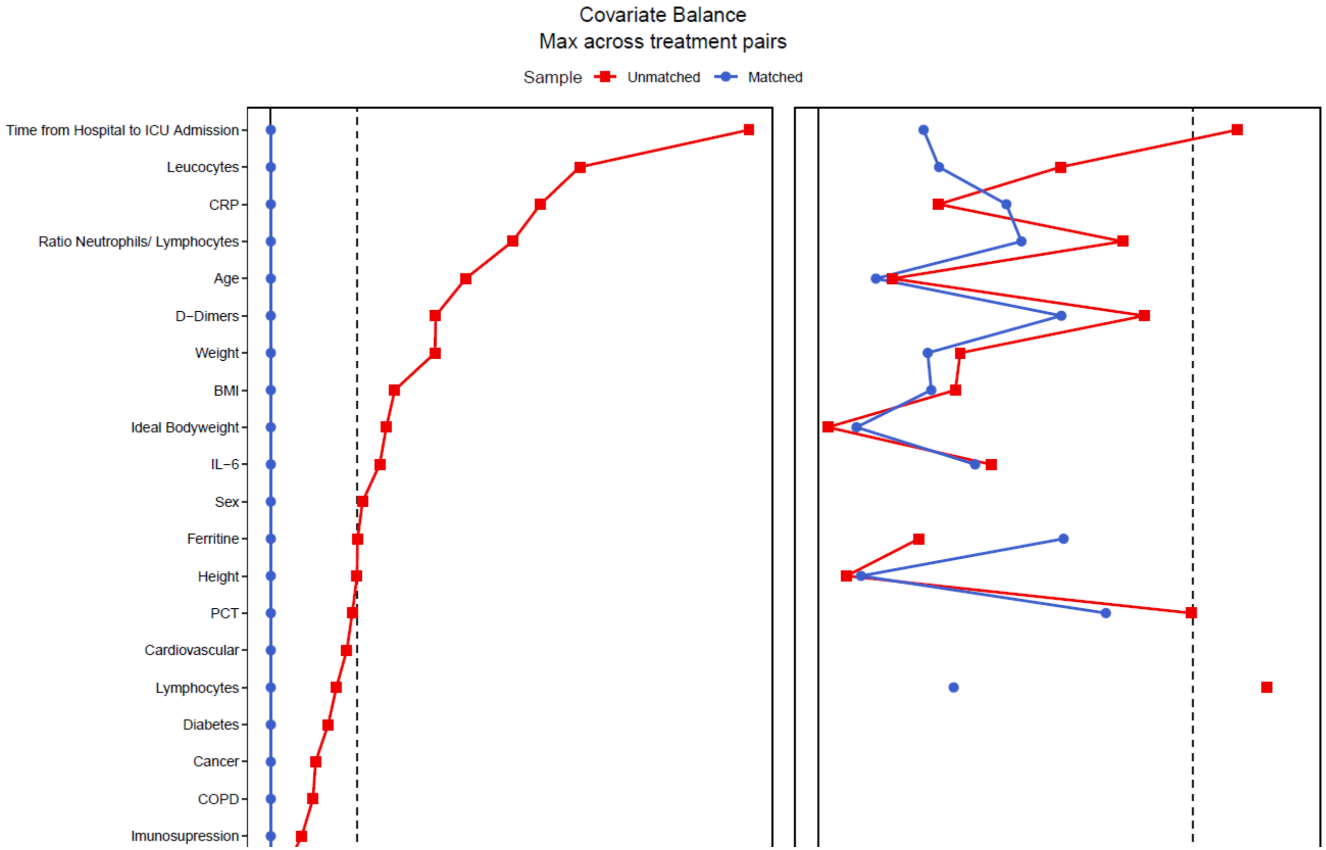

Histograms assessing covariance balancing weight distributions for each tested algorithm. A Gaussian distribution around the 1 without extreme weights is regarded as optimal. Algorithms: classic inverse probability weighting (PS), generalized boosted models (GBM), covariate balancing propensity score weighting (CBPS), nonparametric covariate balancing propensity score weighting (NPCBPS), entropy balancing (EBAL), empirical balancing calibration weighting (EBCW), targeted stable balancing weights using optimization (OPTWEIGHT), Bayesian additive regression trees weighting (BART) and energy balancing (ENERGY).

Difference in maximal standardized mean differences (SMD) and variance ratios (VR) between the unbalanced population (in red) and the *targeted stable balancing weights using optimization covariance* balanced population for each baseline variable, are presented. An SMD value <0.1 represents a negligible difference between group means and a VR <2 a negligible difference between higher-order moments and interactions between groups.

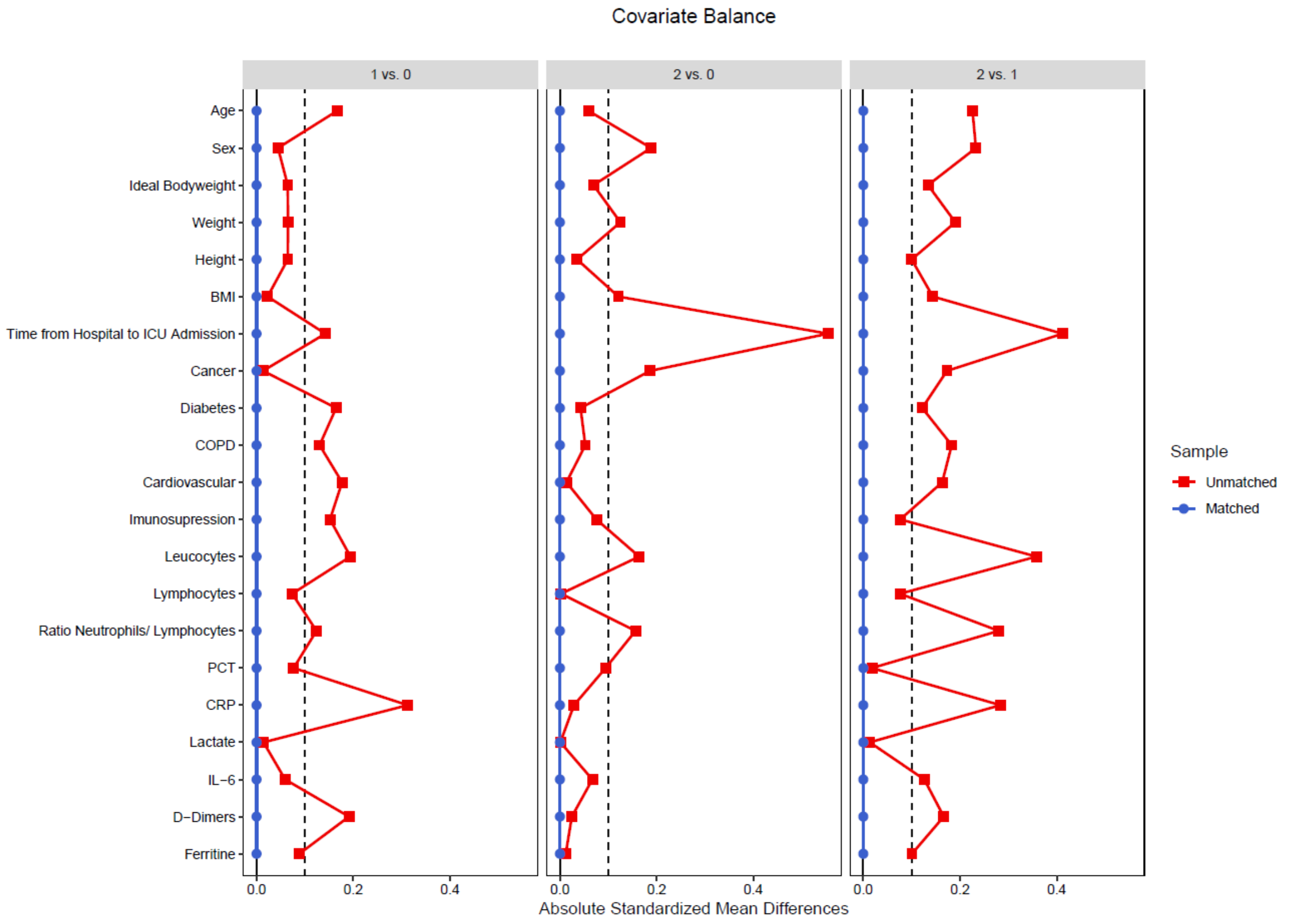

Difference in maximal standardized mean differences (SMD) between the unbalanced population (in red) and the *targeted stable balancing weights using optimization covariance* balanced population for each baseline variable, are presented. An SMD value <0.1 represents a negligible difference between group means. Group 0: oxygen mask, Group 1: high-flow oxygen therapy, Group 2: non-invasive ventilation

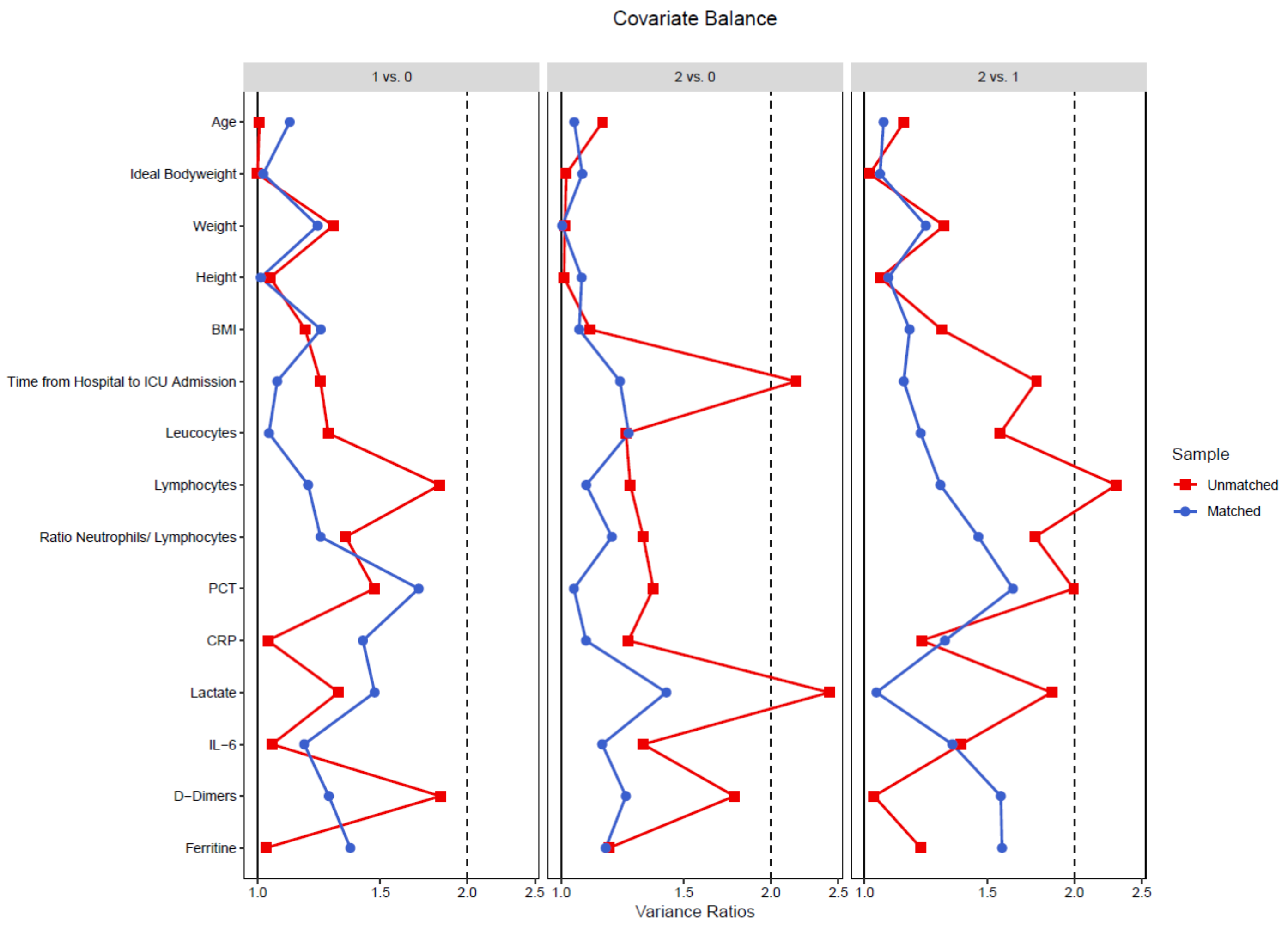

Difference in Variance Ratios (VR) between the unbalanced population (in red) and the *targeted stable balancing weights using optimization covariance* balanced population for each baseline variable, are presented. A VR value <2 represents a negligible difference between higher-order moments and interactions between groups. Group 0: oxygen Mask, Group 1: high flow oxygen therapy, Group 2: non-invasive ventilation

e-Figure 6: Final covariate balancing model – individual variable distributions

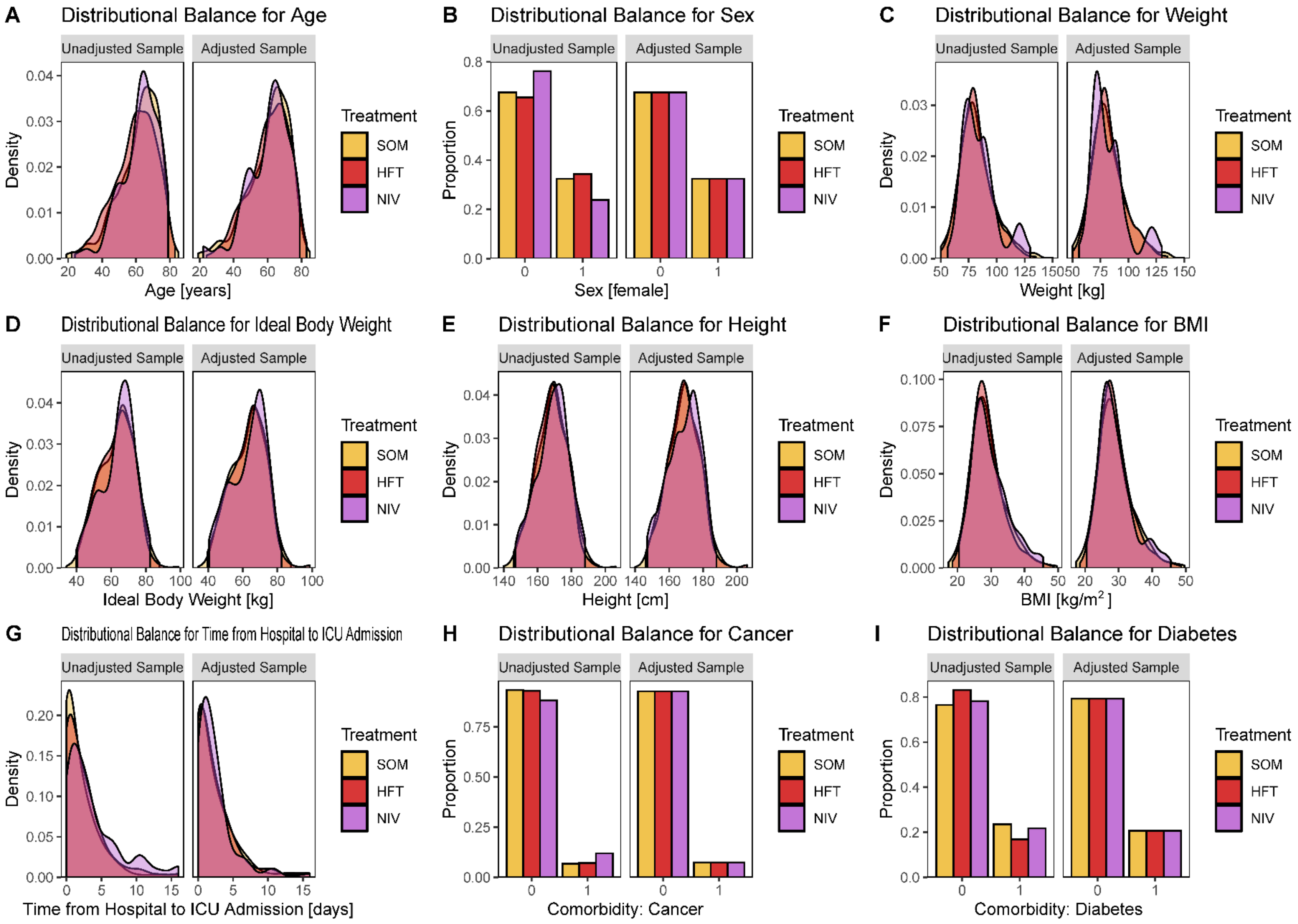

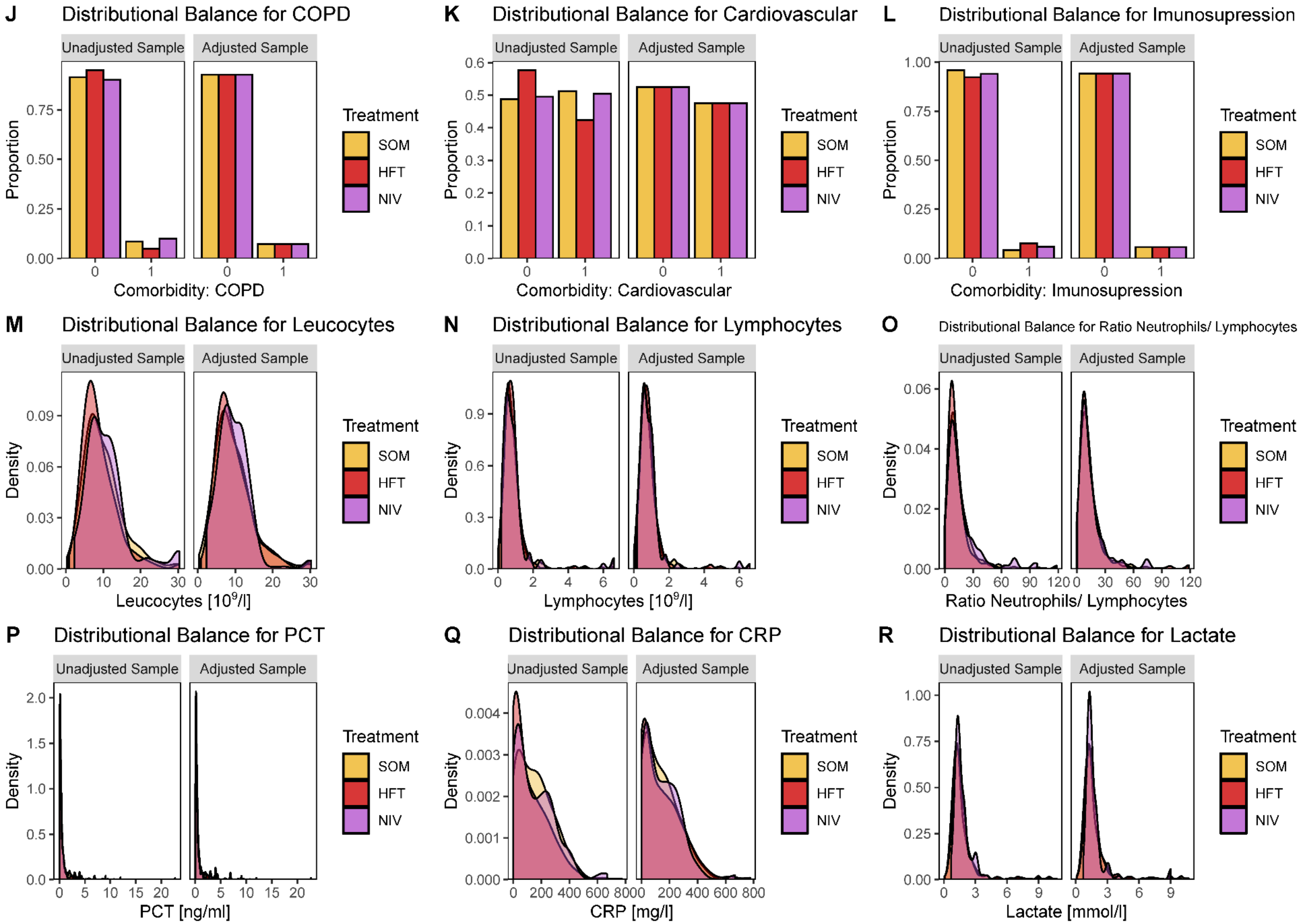
J
Distributional Balance for COPD
Unadjusted Sample
Adjusted Sample
Proportion
Comorbidity: COPD
K
Distributional Balance for Cardiovascular
Unadjusted Sample
Adjusted Sample
Proportion
Comorbidity: Cardiovascular
L
Distributional Balance for Imunosupression
Unadjusted Sample
Adjusted Sample
Proportion
Comorbidity: Imunosupression
M
Distributional Balance for Leucocytes
Unadjusted Sample
Adjusted Sample
Density
Leucocytes [10^9/l]
N
Distributional Balance for Lymphocytes
Unadjusted Sample
Adjusted Sample
Density
Lymphocytes [10^9/l]
O
Distributional Balance for Ratio Neutrophils/ Lymphocytes
Unadjusted Sample
Adjusted Sample
Density
Ratio Neutrophils/ Lymphocytes
P
Distributional Balance for PCT
Unadjusted Sample
Adjusted Sample
Density
PCT [ng/ml]
Q
Distributional Balance for CRP
Unadjusted Sample
Adjusted Sample
Density
CRP [mg/l]
R
Distributional Balance for Lactate
Unadjusted Sample
Adjusted Sample
Density
Lactate [mmol/l]
Treatment
SOM
HFT
NIV

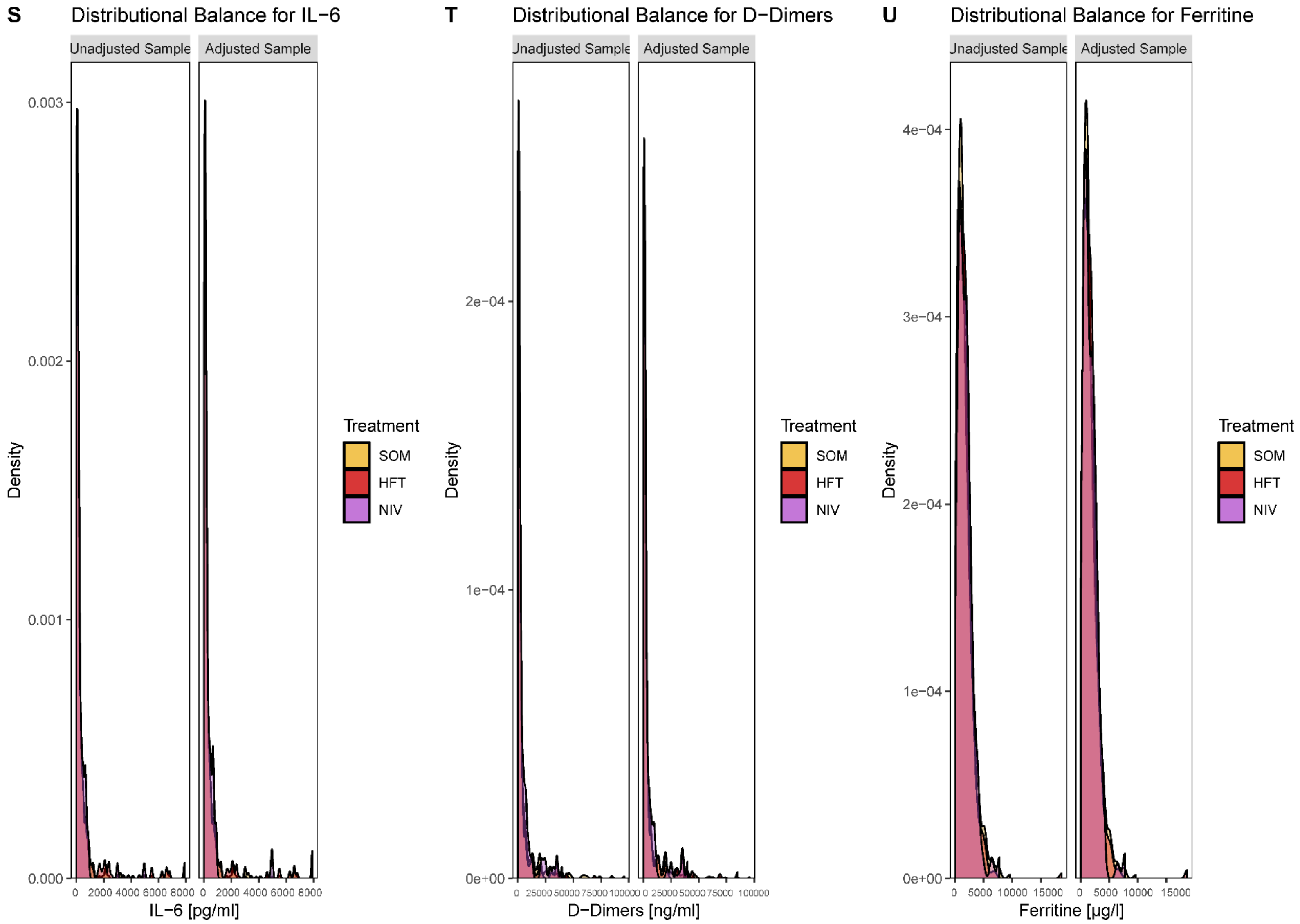

Distribution plots and histograms assessing the differences between the unadjusted and *targeted stable balancing weights using optimization covariance* adjusted population. For categorical variables 1 implies presence of the category and 0 absence of the same. SOM - oxygen mask; HFT – high-flow oxygen therapy; NIV - non-invasive ventilation

e-Figure 7: Final covariate balancing model – model weights

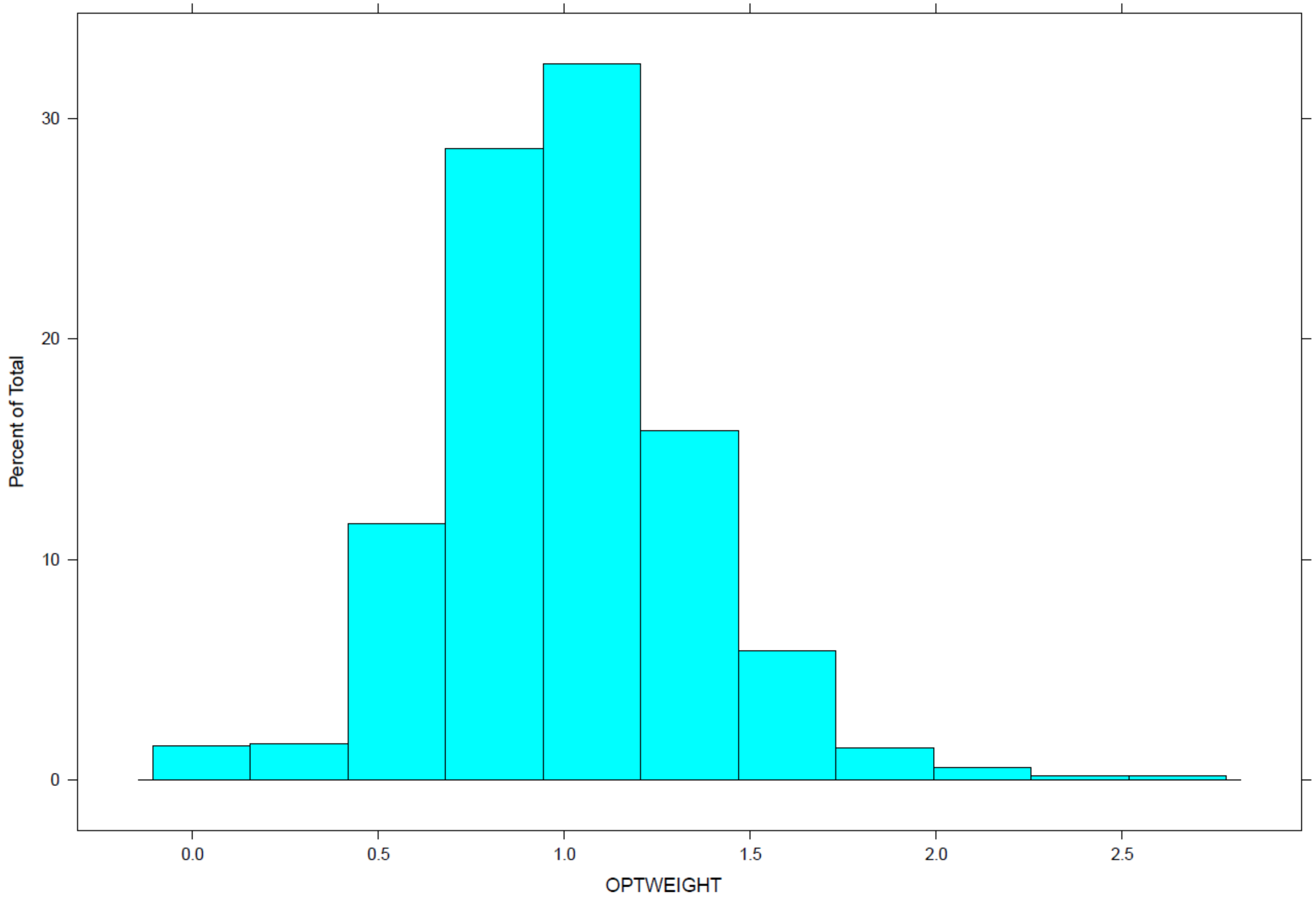

Histogram assessing the covariance balancing weight distribution for the *targeted stable balancing weights using optimization* (OPTWEIGHT) algorithm. A Gaussian distribution around the 1 without extreme weights is regarded as optimal.

e-Figure 8: Static compliance versus time from ICU admission until intubation

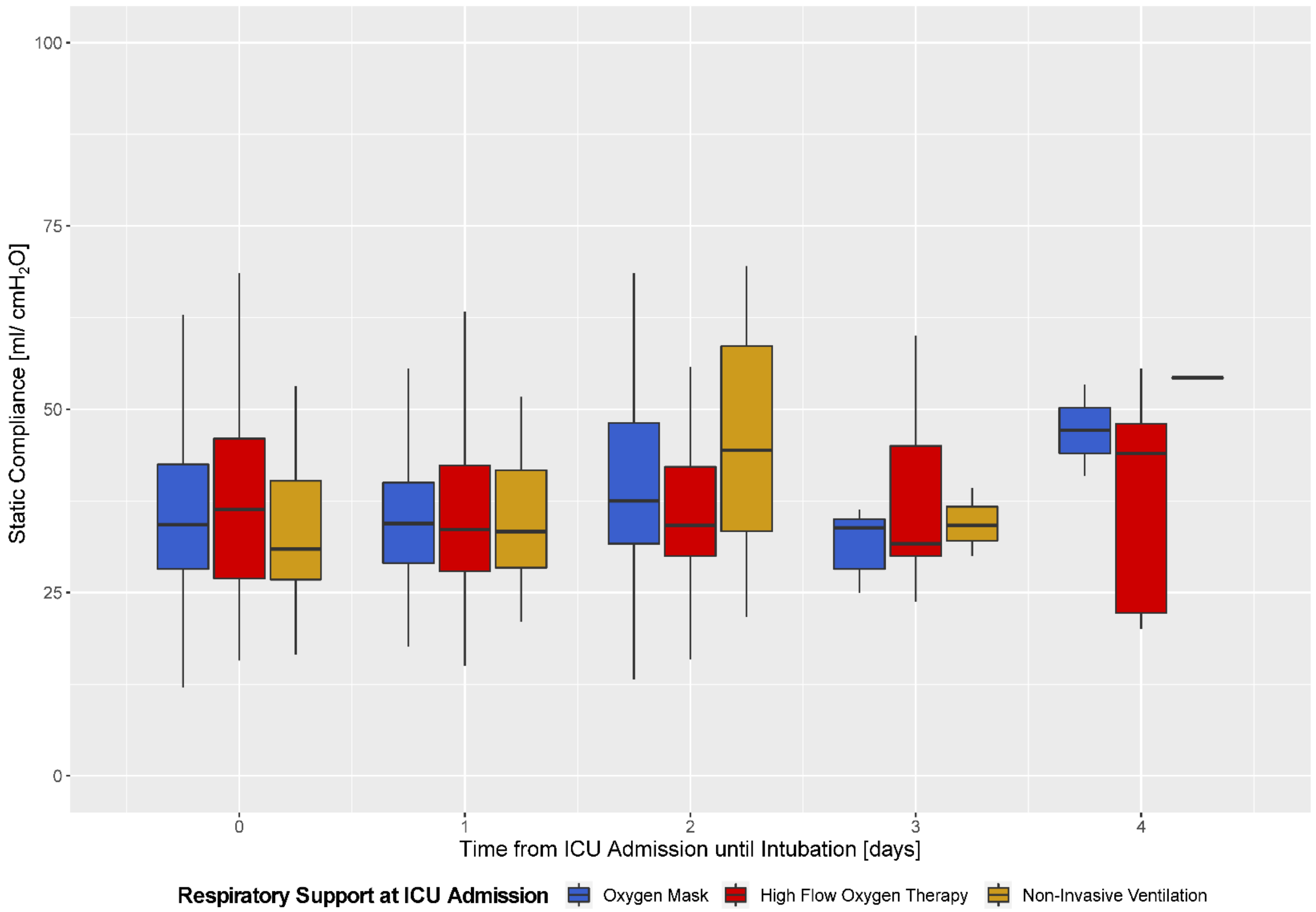

Box-plot assessing the compliance at the moment of intubation dependent on the time between intensive care unit admission and intubation. The plot is stratified by initial respiratory support strategy.

e-Figure 9: Driving pressure versus time from ICU admission until intubation

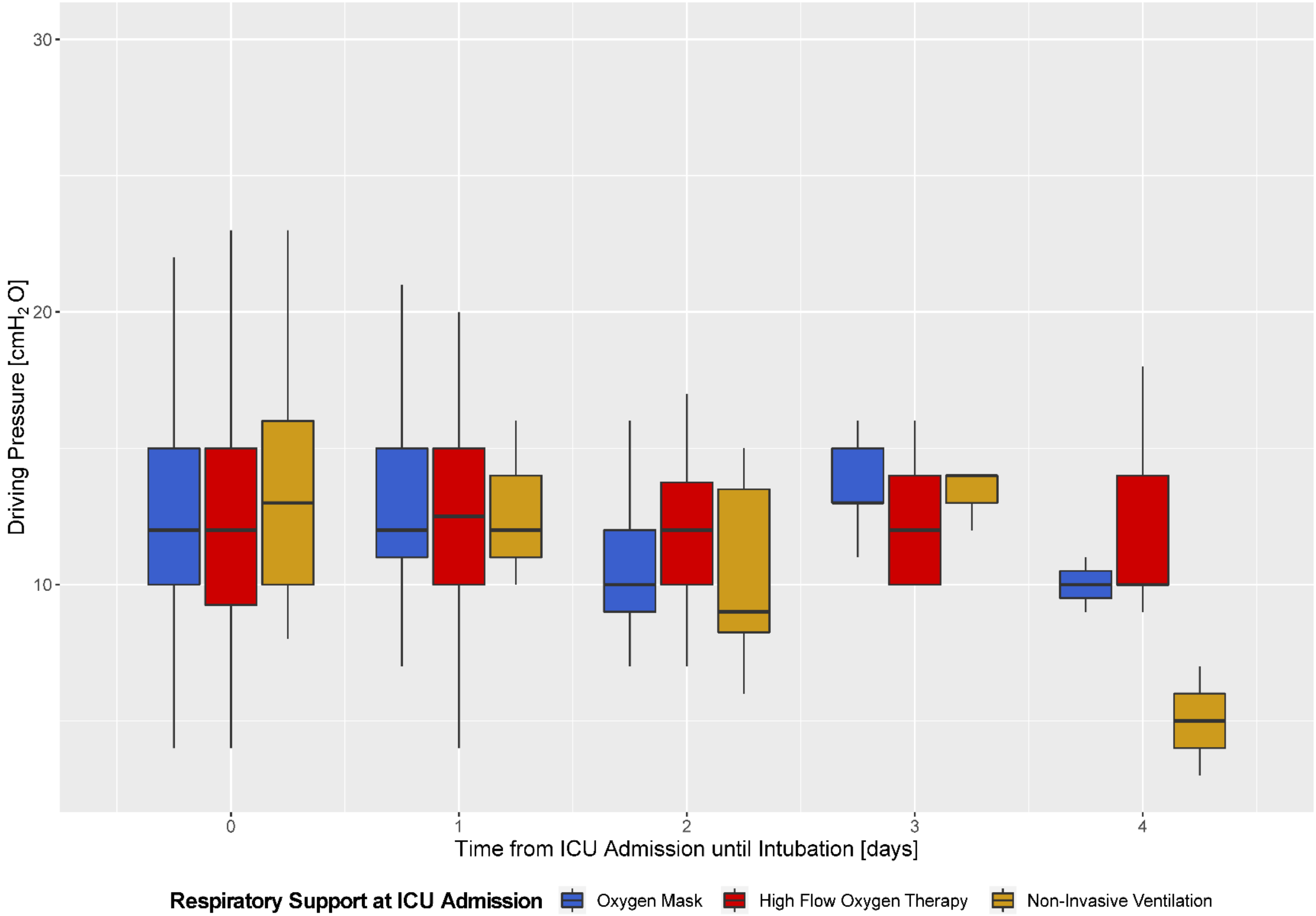

Box-plot assessing the driving pressure at the moment of intubation dependent on the time between intensive care unit admission and intubation. The plot is stratified by initial respiratory support strategy.

e-Figure 10: PaO_2/ fiO_2 ratio versus time from ICU admission until intubation

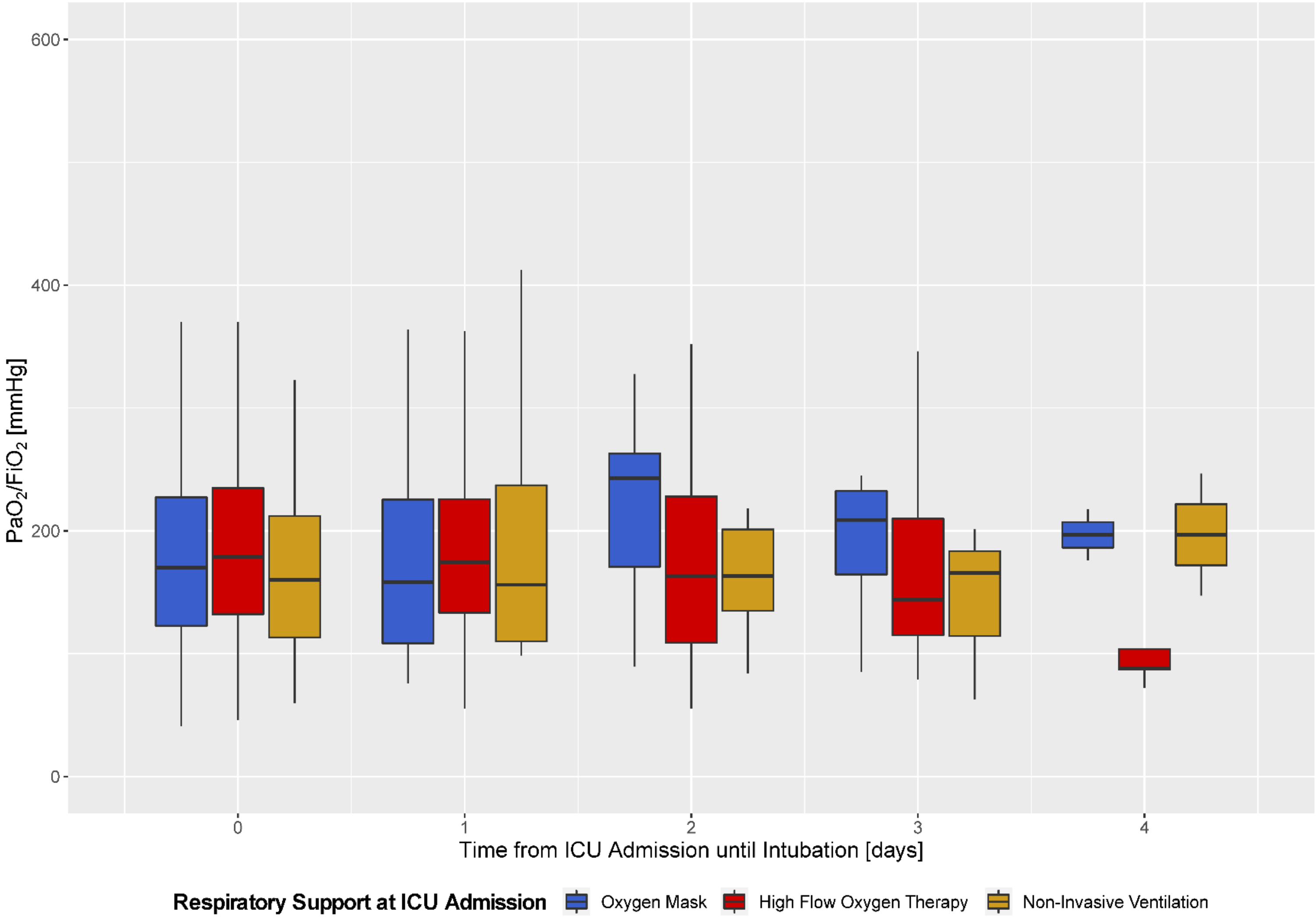

Box-plot assessing the paO_2/ fiO_2 at the moment of intubation dependent on the time between intensive care unit admission and intubation. The plot is stratified by initial respiratory support strategy.

e-Figure 11: Cox time varying model for influence of time until intubation on ICU mortality

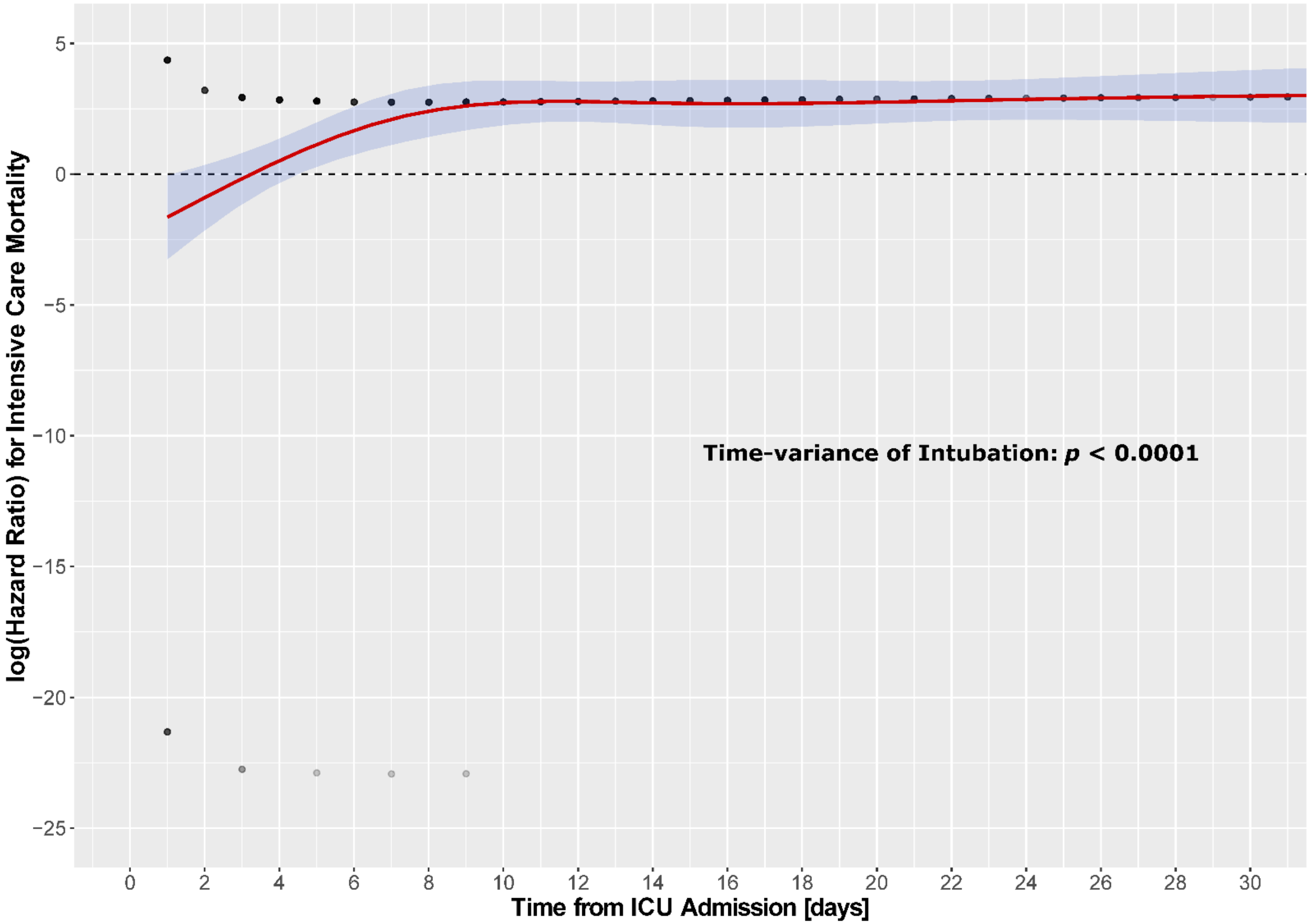

Schoenfeld residuals of a time-varying Cox proportional hazards model assessing the time-dependent effect of intubation on intensive care mortality. Schoenfeld residuals are plotted as dots, intensity of the dots correlates with the number of overlapping individual residuals. Schoenfeld residuals were modelled by a natural cubic spline with 4 knots, depicted as red line. The 95% confidence interval of regression is presented in blue shaded areas. The dotted line represents a hazard ratio of 1.

Schoenfeld residuals can be regarded as the observed mortality hazard for every individual minus the expected hazard, which is defined as the overall estimated mortality hazard by the Cox model. The model shows that the hazard of mortality associated with intubation is not proportional over time. An early intubation, from the time point of intensive care unit admission, was associated with a reduction in the mortality hazard, whereas after about 3 days in the intensive care unit, this protective effect completely disappears and becomes associated with a higher mortality hazard from day 5 on.

<u>e-Figure 12:</u> <u>Multivariable Cox proportional hazards model for in-ICU mortality *(overall study population - covariance balanced)*</u>

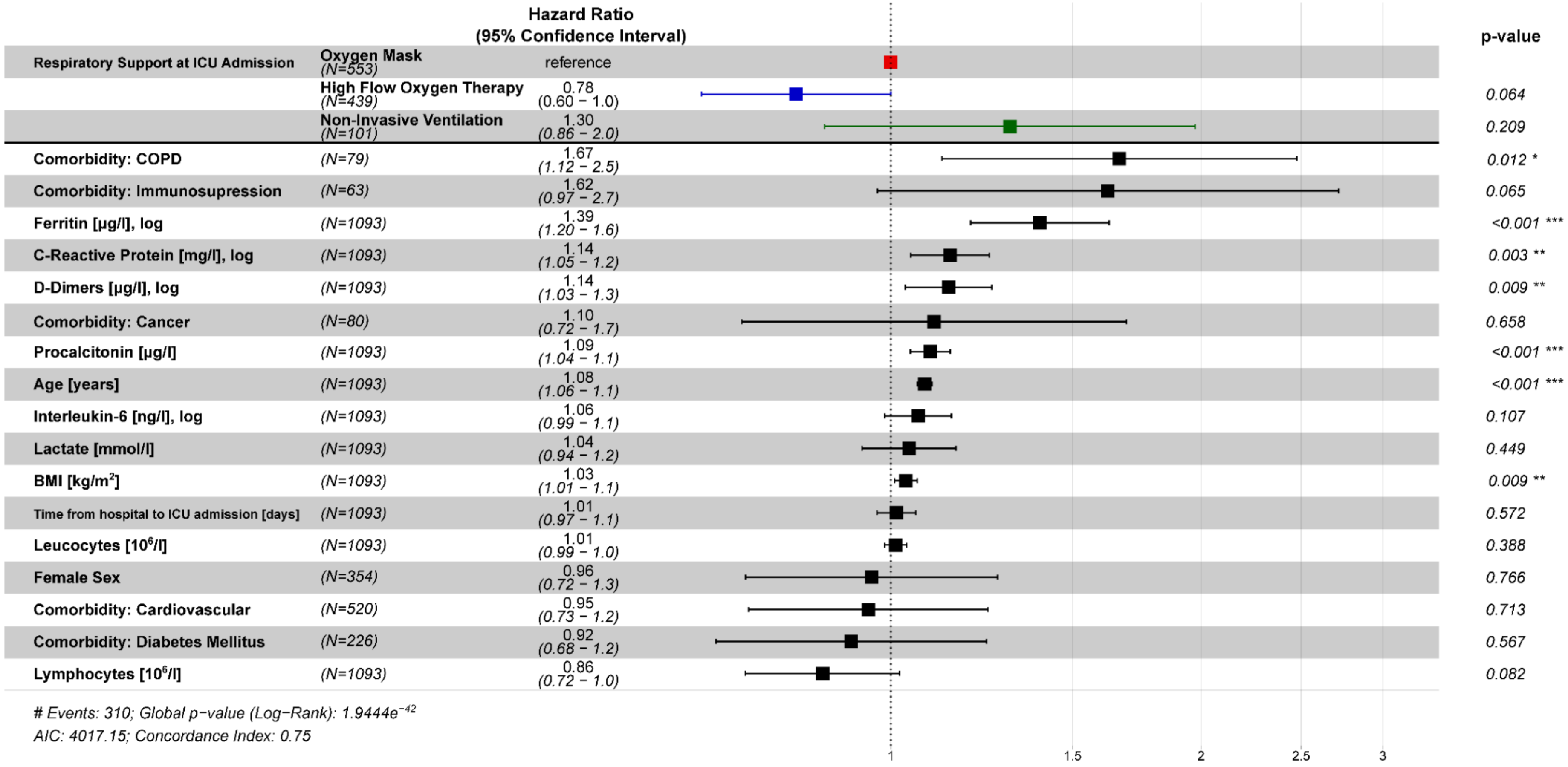

Multivariable Cox proportional hazard model assessing prognostic variables for intensive care mortality and represented in the form of a forest plot. All patients in the covariate balanced population are reflected.

e-Figure 13: Multivariable Cox proportional hazards model for in-ICU mortality *(only intubated - covariance balanced)*

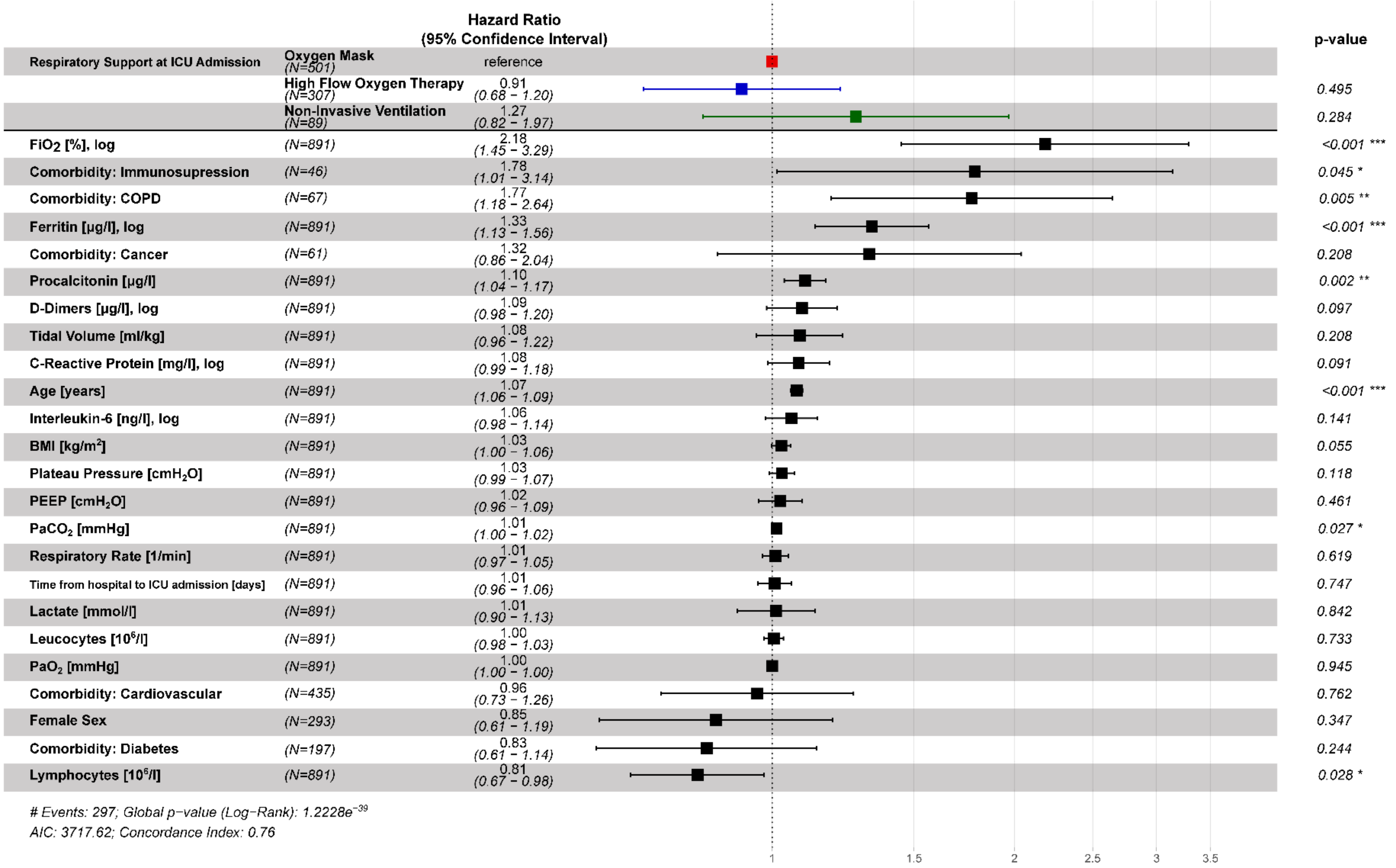

Multivariable Cox proportional hazard model assessing prognostic variables for intensive care mortality and represented in the form of a forest plot. Only intubated patients in the covariate balanced population are reflected.

e-Figure 14: Kaplan-Meier curve for ICU mortality – compliance vs. D-dimers

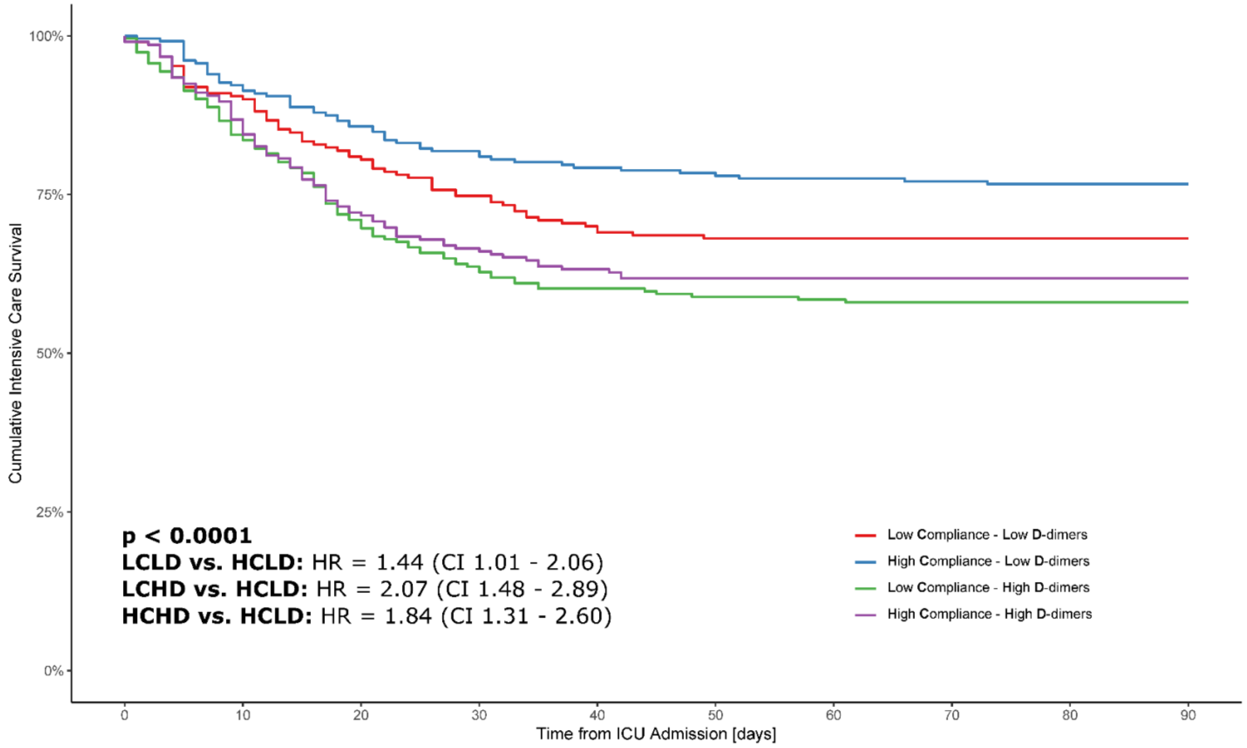

	0	10	20	30	40	50	60	70	80	90
LCLD	210	190	170	157	147	143	143	143	143	143
HCLD	231	213	198	189	183	181	179	178	177	177
LCHD	231	195	164	147	139	136	135	134	134	134
HCHD	212	184	153	141	134	131	131	131	131	131

Kaplan-Meier curve of the cumulative intensive care survival stratified by the interaction between compliance and D-dimers. Low and High definitions are chosen based on a median cut-off. p-values are calculated by means of the log-rank test. Hazard ratios (HR) are computed by means of a Cox proportional hazard model and employ the high compliance, high D-dimer group as reference, 95% confidence intervals (CI) are given in parentheses. The underlying table presents the patients at risk per time point. LCLD: Low compliance – Low D-dimers, HCLD: High compliance – Low D-dimers, LCHD: Low compliance – High D-dimers, HCHD: High compliance – High D-dimers.

e-Figure 15: Kaplan-Meier curve for ICU mortality in unbalanced population

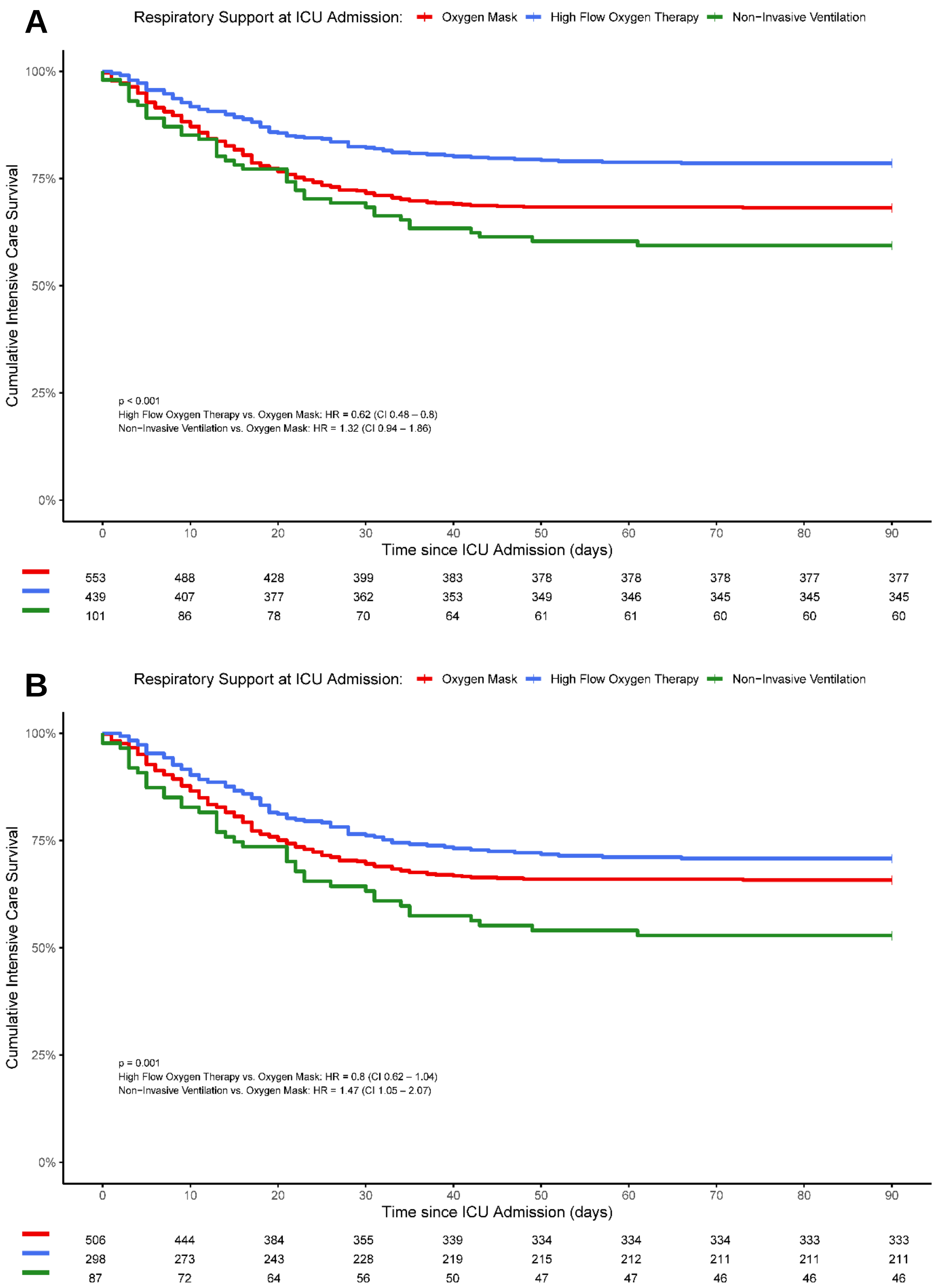

(red)	553	488	428	399	383	378	378	378	377	377
(blue)	439	407	377	362	353	349	346	345	345	345
(green)	101	86	78	70	64	61	61	60	60	60

(red)	506	444	384	355	339	334	334	334	333	333
(blue)	298	273	243	228	219	215	212	211	211	211
(green)	87	72	64	56	50	47	47	46	46	46

Kaplan-Meier curve of the cumulative intensive care survival stratified by initial respiratory support strategy at intensive care unit admission. Subplot (A) refers to all patients included in the analysis, whereas in (B) only patients progressing towards intubation and invasive mechanical ventilation are considered. p-values are calculated by means of the log-rank test. Hazard ratios (HR) are computed by means of a Cox proportional hazard model and assesses the risk of intensive care unit mortality in the high flow oxygen therapy and non-invasive ventilation groups using the oxygen mask group as reference, 95% confidence intervals (CI) are given in parentheses. The underlying table presents the patients at risk per time point. Patients are assessed without covariance balancing.

e-Table 5: Cox mixed-effects model with between-center random effects term (*unbalanced*)

In order to avoid excessive unbalancing of the mixed-effects model due to centers not having treated patients with all three non-invasive respiratory support strategies, only centers having treated patients with oxygen mask, high-flow oxygen therapy and non-invasive ventilation were regarded in this sensitivity analysis.

Centers included in sensitivity analysis: 19/ 26

Patients included in sensitivity analysis: 970/ 1093

90-day Hazard of Intubation

	Variance	Standard Deviation	
**Random Effect - Center**			
Intercept	0.4079	0.1664	
	**Estimate**	**95% Confidence Interval**	**p**
**Fixed Effects**			
Oxygen Mask	1.0	1.0 – 1.0	Ref.
High-Flow Oxygen Therapy	0.3668	0.2965 - 0.4537	<0.001
Non-Invasive Ventilation	0.6514	0.4966 - 0.8545	0.002

90-day Hazard of ICU Mortality

	Variance	Standard Deviation	
**Random Effect - Center**			
Intercept	0.3405	0.1159	
	**Estimate**	**95% Confidence Interval**	**p**
**Fixed Effects**			
Oxygen Mask	1.0	1.0 – 1.0	Ref.
High-Flow Oxygen Therapy	0.7111	0.5113 - 0.9888	0.043
Non-Invasive Ventilation	1.5854	1.071 - 2.3475	0.021

90-day Hazard of ICU Mortality (*only intubated patients*)

	Variance	Standard Deviation	
**Random Effect - Center**			
Intercept	0.3754	0.1409	
	**Estimate**	**95% Confidence Interval**	**p**
**Fixed Effects**			
Oxygen Mask	1.0	1.0 – 1.0	Ref.
High-Flow Oxygen Therapy	1.0309	0.7314 - 1.4528	0.860
Non-Invasive Ventilation	1.8214	1.2186 - 2.7224	0.004

e-Figure 16: Multivariable Cox proportional hazards model for in-ICU mortality *(overall study population - unbalanced)*

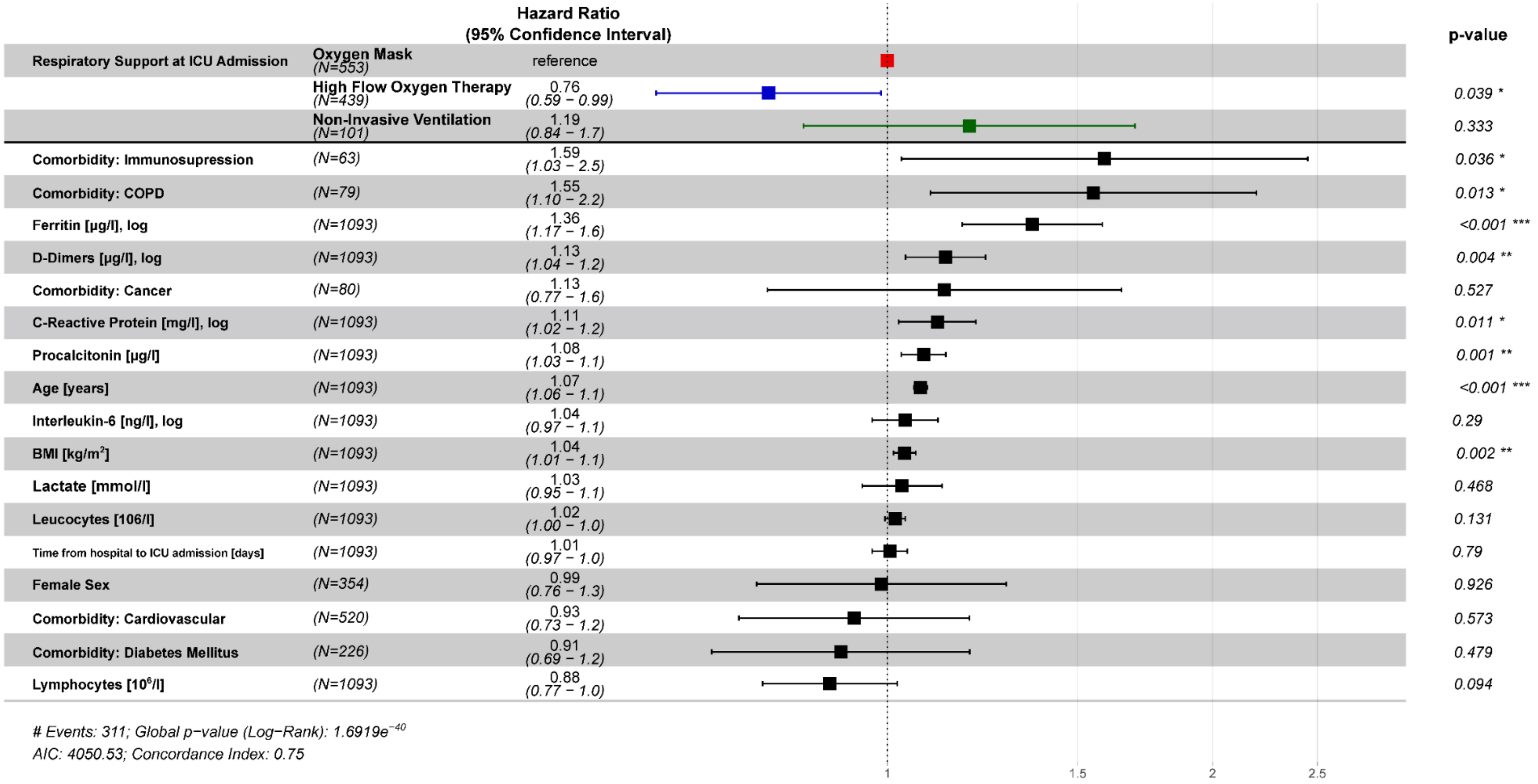

Multivariable Cox proportional hazard model assessing prognostic variables for intensive care mortality and represented in the form of a forest plot. All patients in the unbalanced population are reflected.

e-Figure 17: Multivariable Cox proportional hazards model for in-ICU mortality *(only intubated - unbalanced)*

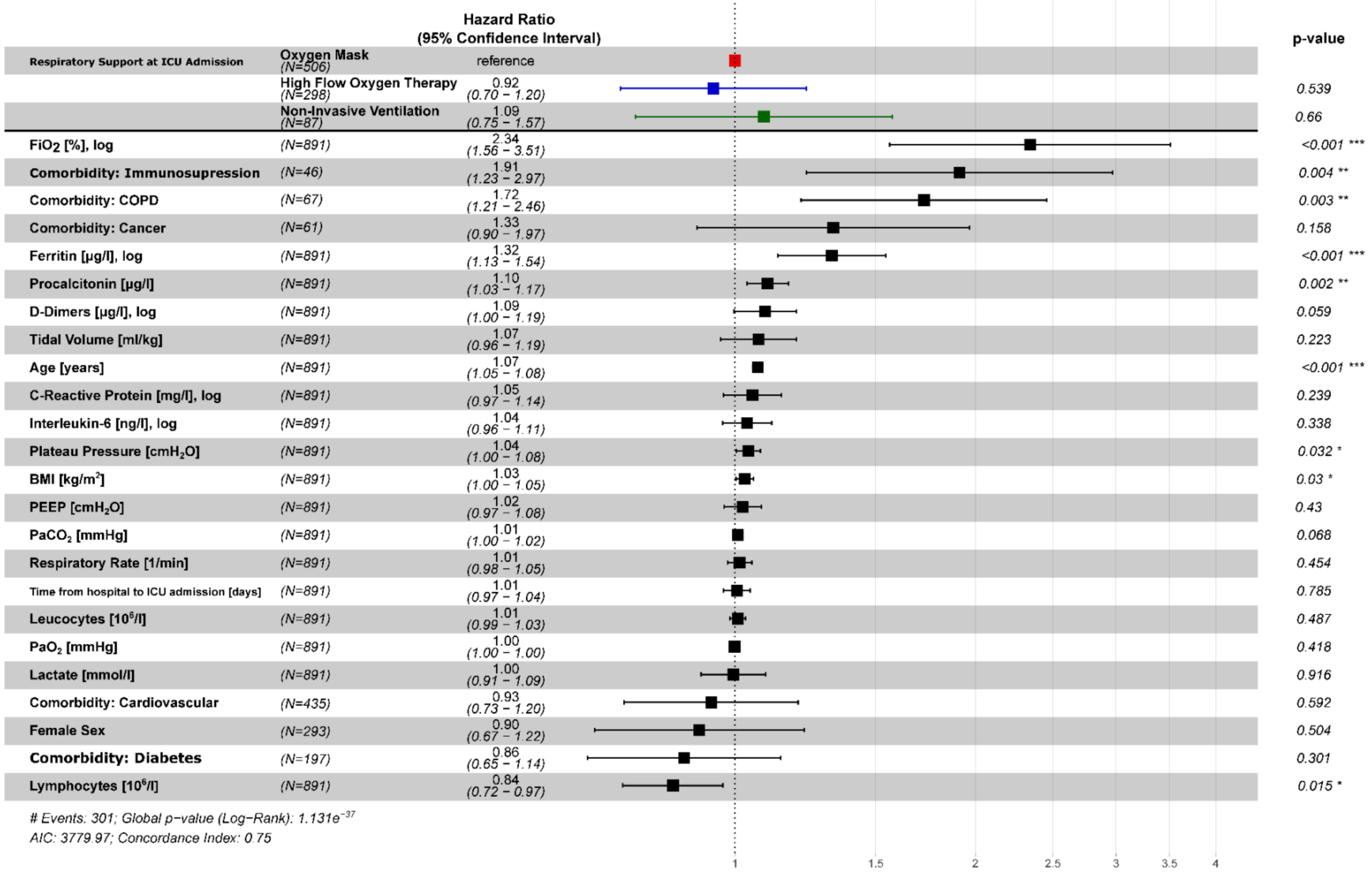

Multivariable Cox proportional hazard model assessing prognostic variables for intensive care mortality and represented in the form of a forest plot. Only intubated patients in the unbalanced population are reflected.

e-Figure 18: Multivariable Cox mixed-effects model with between-center random effects term for in-ICU mortality *(overall study population – unbalanced)*

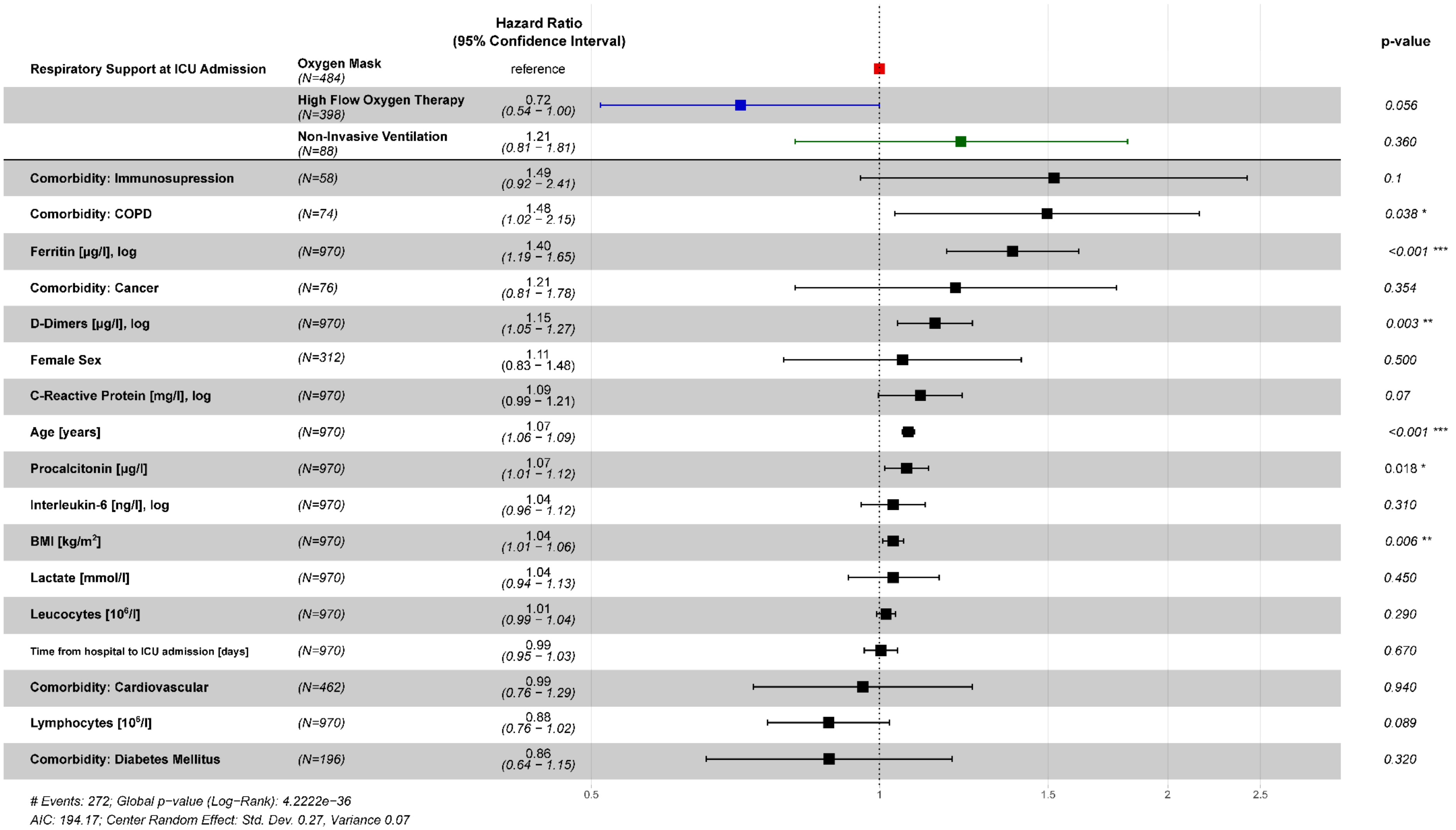

Multivariable Cox mixed-effects model with between-center random effects term assessing prognostic variables for intensive care mortality presented in the form of a forest plot. In order to avoid excessive unbalancing of the mixed-effects only centers having treated patients with all three non-invasive respiratory support strategies were regarded in this sensitivity analysis. Centers included in sensitivity analysis: 19/ 26. Patients included in sensitivity analysis: 970/ 1093

e-Figure 19: Multivariable Cox mixed-effects model with between-center random effects term for in-ICU mortality *(only intubated - unbalanced)*

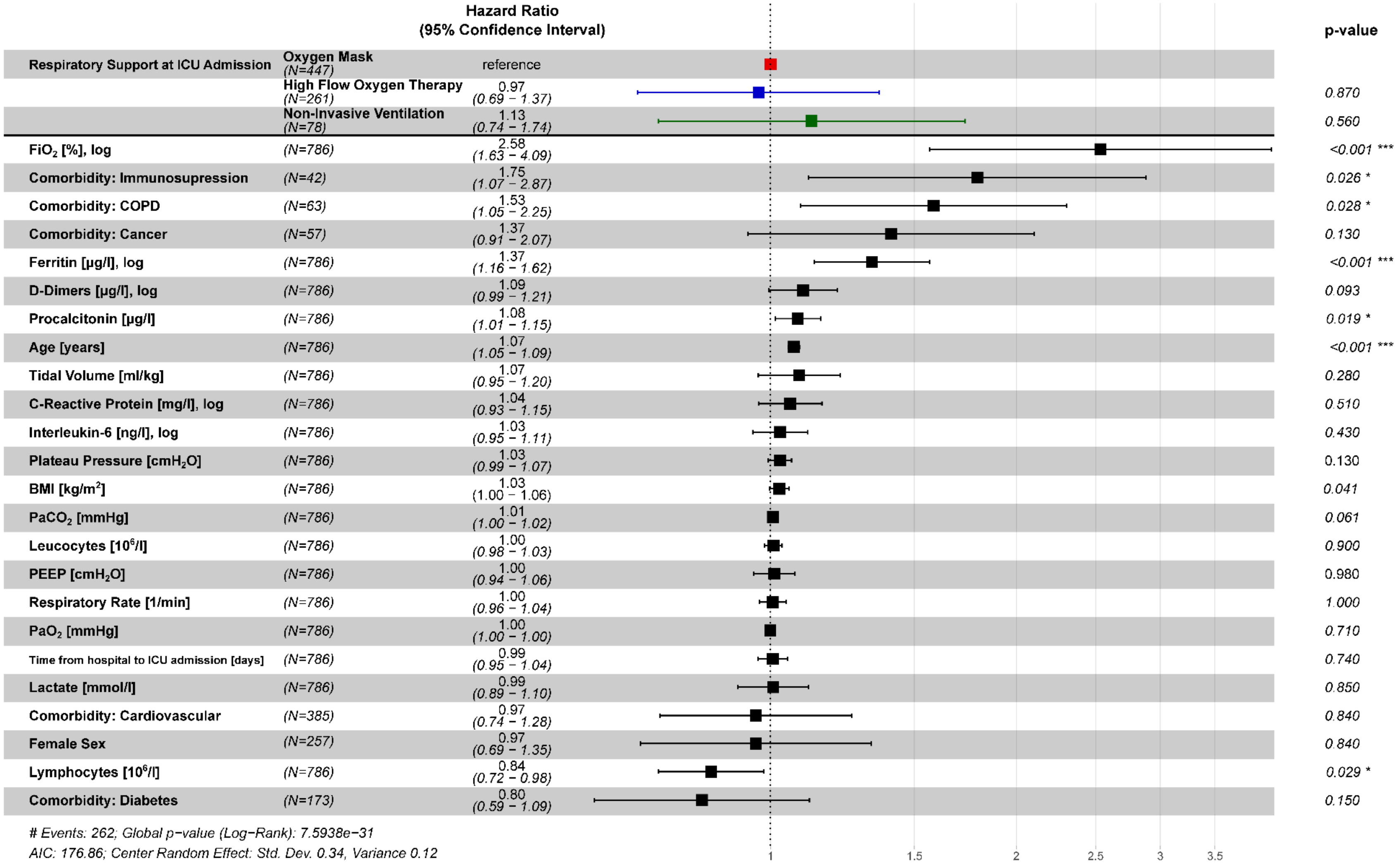

Multivariable Cox mixed-effects model with between-center random effects term assessing prognostic variables for intensive care mortality presented in the form of a forest plot. In order to avoid excessive unbalancing of the mixed-effects only centers having treated patients with all three non-invasive respiratory support strategies were regarded in this sensitivity analysis. Centers included in sensitivity analysis: 19/ 26. Patients included in sensitivity analysis: 786/ 891

e-Appendix 4: Formulas

- Ventilatory Ratio [26]

$$\boldsymbol{Ventilatory\ Ratio} = \frac{\boldsymbol{Minute\ Ventilation_{measured}} \left[\frac{\boldsymbol{mL}}{\boldsymbol{min}}\right] \times \boldsymbol{PaCO_{2\ measured}}\ [\boldsymbol{mmHg}]}{\boldsymbol{Minute\ Ventilation_{predicted}} \left[\frac{\boldsymbol{mL}}{\boldsymbol{min}}\right] \times \boldsymbol{PaCO_{2\ ideal}}\ [\boldsymbol{mmHg}]}$$

$$Minute\ Ventilation_{predicted} = Predicted\ Body\ Weight\ \times 100\ \left[\frac{mL}{min}\right]$$

$$PaCO_{2\ ideal} = 37.5\ mmHg$$

- Estimated Physiological Dead-Space Fraction – Unadjusted Harris-Benedict formula [27]

$$\boldsymbol{Estimated\ Physiological\ Dead\ Space} = \boldsymbol{1} - \frac{\boldsymbol{0.863} \times \boldsymbol{\dot{V}CO_2}}{\boldsymbol{Respiratory\ Rate}\ [\boldsymbol{bpm}] \times \boldsymbol{Tidal\ Volume}\ [\boldsymbol{L}] \times \boldsymbol{PaCO_{2\ measured}}\ [\boldsymbol{mmHg}]}$$

$$\dot{V}CO_2 = \frac{Resting\ Energy\ Expenditure}{\frac{5.616}{0.8}} + 1.584$$

$$Resting\ Enery\ Expenditure_{Unadjusted\ Harris-Benedict\ Estimate-Male} = 66.473\ +\ 13.752\ \times Weight\ [kg] + 5.003 \times\ Height\ [cm] -\ 6.755 \times\ Age\ [years]$$

$$Resting\ Enery\ Expenditure_{Unadjusted\ Harris-Benedict\ Estimate-Female} = 66.473\ +\ 13.752\ \times Weight\ [kg] + 5.003 \times\ Height\ [cm] -\ 6.755 \times\ Age\ [years]$$

References

1. Potthoff RF, Tudor GE, Pieper KS, Hasselblad V: **Can one assess whether missing data are missing at random in medical studies?** *Stat Methods Med Res* 2006, **15**(3):213-234.
2. van Buuren S: **Flexible Imputation of Missing Data**, 2nd ed. edn: Chapman and Hall/CRC; 2018.
3. Qu Y, Lipkovich I: **Propensity score estimation with missing values using a multiple imputation missingness pattern (MIMP) approach**. *Stat Med* 2009, **28**(9):1402-1414.
4. Jakobsen JC, Gluud C, Wetterslev J, Winkel P: **When and how should multiple imputation be used for handling missing data in randomised clinical trials – a practical guide with flowcharts**. *BMC Medical Research Methodology* 2017, **17**(1):162.
5. Madley-Dowd P, Hughes R, Tilling K, Heron J: **The proportion of missing data should not be used to guide decisions on multiple imputation**. *Journal of Clinical Epidemiology* 2019, **110**:63-73.
6. van Ginkel JR, Linting M, Rippe RCA, van der Voort A: **Rebutting Existing Misconceptions About Multiple Imputation as a Method for Handling Missing Data**. *Journal of Personality Assessment* 2020, **102**(3):297-308.
7. Austin PC, White IR, Lee DS, van Buuren S: **Missing Data in Clinical Research: A Tutorial on Multiple Imputation**. *Canadian Journal of Cardiology* 2021.
8. Qu Y, Lipkovich I: **Propensity score estimation with missing values using a multiple imputation missingness pattern (MIMP) approach**. *Statistics in Medicine* 2009, **28**(9):1402-1414.
9. Potthoff RF, Tudor GE, Pieper KS, Hasselblad V: **Can one assess whether missing data are missing at random in medical studies?** *Statistical Methods in Medical Research* 2006, **15**(3):213-234.

10. Rosenbaum PR, Rubin DB: **The central role of the propensity score in observational studies for causal effects**. *Biometrika* 1983, **70**(1):41-55.

11. Rubin DB: **Should observational studies be designed to allow lack of balance in covariate distributions across treatment groups?** *Statistics in Medicine* 2009, **28**(9):1420-1423.

12. Rubin DB: **Using Propensity Scores to Help Design Observational Studies: Application to the Tobacco Litigation**. *Health Services and Outcomes Research Methodology* 2001, **2**(3):169-188.

13. Elizabeth AS: **Matching Methods for Causal Inference: A Review and a Look Forward**. *Statistical Science* 2010, **25**(1):1-21.

14. Cooper GF, Glymour C: **On the Impossibility of Inferring Causation from Association without Background Knowledge**. In: *Computation, Causation, and Discovery*. AAAI Press; 1999: 0.

15. Rosenbaum PR: **Model-Based Direct Adjustment**. *Journal of the American Statistical Association* 1987, **82**(398):387-394.

16. Ridgeway G: **Generalized Boosted Models: A Guide to the GBM Package**. *Compute* 2005, **1**:1-12.

17. Imai K, Ratkovic M: **Covariate balancing propensity score**. *Journal of the Royal Statistical Society: Series B (Statistical Methodology)* 2014, **76**(1):243-263.

18. Hainmueller J: **Entropy Balancing for Causal Effects: A Multivariate Reweighting Method to Produce Balanced Samples in Observational Studies**. *Political Analysis* 2011, **20**.

19. Chan KCG, Yam SCP, Zhang Z: **Globally efficient non-parametric inference of average treatment effects by empirical balancing calibration weighting**. *Journal of the Royal Statistical Society: Series B (Statistical Methodology)* 2016, **78**(3):673-700.

20. Zubizarreta J: **Stable Weights that Balance Covariates for Estimation With Incomplete Outcome Data**. *Journal of the American Statistical Association* 2015, **110**:0-0.

21. Chipman HA, George EI, McCulloch RE: **Bart: Bayesian Additive Regression Trees**. *The Annals of Applied Statistics* 2010, **4**(1):266-298.

22. Huling J, Mak S: **Energy Balancing of Covariate Distributions**. *arXiv: Methodology* 2020.

23. Austin PC, Stuart EA: **Moving towards best practice when using inverse probability of treatment weighting (IPTW) using the propensity score to estimate causal treatment effects in observational studies**. *Stat Med* 2015, **34**(28):3661-3679.

24. Normand S-LT, Landrum MB, Guadagnoli E, Ayanian JZ, Ryan TJ, Cleary PD, McNeil BJ: **Validating recommendations for coronary angiography following acute myocardial infarction in the elderly: A matched analysis using propensity scores**. *Journal of Clinical Epidemiology* 2001, **54**(4):387-398.

25. Linden A, Samuels SJ: **Using balance statistics to determine the optimal number of controls in matching studies**. *Journal of Evaluation in Clinical Practice* 2013, **19**(5):968-975.

26. Sinha P, Calfee CS, Beitler JR, Soni N, Ho K, Matthay MA, et al. **Physiologic Analysis and Clinical Performance of the Ventilatory Ratio in Acute Respiratory Distress Syndrome**. Am J Respir Crit Care Med. 2019;199(3):333-41.

27. Morales-Quinteros L, Neto AS, Artigas A, Blanch L, Botta M, Kaufman DA, et al. **Dead space estimates may not be independently associated with 28-day mortality in COVID-19 ARDS**. Critical Care. 2021;25(1):171.
